# DoEstRare: A statistical test to identify local enrichments in rare genomic variants associated with disease

**DOI:** 10.1371/journal.pone.0179364

**Published:** 2017-07-24

**Authors:** Elodie Persyn, Matilde Karakachoff, Solena Le Scouarnec, Camille Le Clézio, Dominique Campion, French Exome Consortium, Jean-Jacques Schott, Richard Redon, Lise Bellanger, Christian Dina

**Affiliations:** 1 INSERM, CNRS, UNIV Nantes, l’institut du thorax, Nantes, France; 2 CHU Nantes, l’institut du thorax, Nantes, France; 3 Inserm U1079, Rouen University, Normandy Center for Genomic Medicine and Personalized Medicine, Normandy University, Rouen, France; 4 Laboratoire de Mathématiques Jean Leray, UMR CNRS 6629, Nantes, France; Columbia University Medical Center, UNITED STATES

## Abstract

Next-generation sequencing technologies made it possible to assay the effect of rare variants on complex diseases. As an extension of the “common disease-common variant” paradigm, rare variant studies are necessary to get a more complete insight into the genetic architecture of human traits. Association studies of these rare variations show new challenges in terms of statistical analysis. Due to their low frequency, rare variants must be tested by groups. This approach is then hindered by the fact that an unknown proportion of the variants could be neutral. The risk level of a rare variation may be determined by its impact but also by its position in the protein sequence. More generally, the molecular mechanisms underlying the disease architecture may involve specific protein domains or inter-genic regulatory regions. While a large variety of methods are optimizing functionality weights for each single marker, few evaluate variant position differences between cases and controls. Here, we propose a test called DoEstRare, which aims to simultaneously detect clusters of disease risk variants and global allele frequency differences in genomic regions. This test estimates, for cases and controls, variant position densities in the genetic region by a kernel method, weighted by a function of allele frequencies. We compared DoEstRare with previously published strategies through simulation studies as well as re-analysis of real datasets. Based on simulation under various scenarios, DoEstRare was the sole to consistently show highest performance, in terms of type I error and power both when variants were clustered or not. DoEstRare was also applied to Brugada syndrome and early-onset Alzheimer’s disease data and provided complementary results to other existing tests. DoEstRare, by integrating variant position information, gives new opportunities to explain disease susceptibility. DoEstRare is implemented in a user-friendly R package.

## Introduction

Genome-wide association studies (GWASs) have identified numerous common haplotypes associated with a wide variety of complex diseases [1]. However these common variants often present low effects on disease susceptibility and do not explain the whole heritability [2]. Rare variants with stronger effects may explain, among other factors, the missing heritability [3]. These variants are defined with a minor allele frequency (MAF) often arbitrarily set between 0.1% and 1%, depending on the disease prevalence.

The huge advances in genome sequencing are now enabling association studies on rare variants. However single-marker tests (consisting in testing each variant individually) are not suitable in the context of low-frequency alleles, for which immoderately large sample sizes would be required to obtain sufficient power to detect association signals. Many specific statistical methods have thus been developed to test the association between complex diseases and groups of rare variants [4–14]. For efficiency reasons, groups of rare variants often correspond to gene coding sequences, which are biological units easy of interpretation.

One challenge is to deal with the heterogeneous nature of genetic variants. Indeed groups of rare variants are likely to include a non-negligible proportion of neutral variants and causal variants with various effect sizes. Different strategies have been adopted to detect an association in the presence of neutral variants, such as keeping only putative deleterious variants with an adaptive method [7,14] or using continuous weights based on functional potential [6,8,11,12]. It is also recognized that all positions are not equal, corresponding to various protein domains (within a gene) or putative functionality (genome). For instance, Robertson et al. (2003) found that pathogenic mutations are localized in various domains of the *FLNA* gene, causing diverse congenital malformations [15]. Only a few tests take also into account genetic positions, in order to detect clusters of disease-risk variants residing within specific domains of given proteins [16–21].

We developed a new statistical test, named DoEstRare for “Density-oriented Estimation for Rare variant positions”, to detect both global enrichment in rare alleles and localized clusters of disease-risk rare variants (DRVs), by integrating position information. The DoEstRare statistic consists in comparing simultaneously the mutation position densities, estimated by kernel method, and the overall average allele frequencies between cases and controls. To better discriminate neutral from causal variants (deleterious or protective), we incorporated a weight system in the computation of average allele frequencies. A similar approach was used in the Kernel-Based Adaptive Cluster (KBAC) test [8], on multi-locus genotypes.

To assess the performance of DoEstRare, we compared its power and type I error to other existing tests by analyzing simulated data. We conducted simulations, based on the backward coalescent model implemented in the COSI program [22], under different scenarios varying the position distribution of DRVs. We considered three main scenarios: in the first scenario DRVs are uniformly distributed on the gene, in the second and third scenarios DRVs are respectively clustered in one and two areas. We also varied the proportion of causal variants which is linked to the window sizes of clustered areas. From these simulations we show that our test is among the most powerful statistical tests and perform even better with the presence of one cluster of DRVs.

We also applied association tests based on rare alleles on two real datasets to assess the consistency between significance results and evaluate the properties of our test in real settings. The studied pathologies were Brugada syndrome (BrS), with data from Le Scouarnec et al. (2015) [23] and early-onset Alzheimer’s disease (EOAD), from Nicolas et al. (2015) [24]. Interestingly, we show that DoEstRare provides slightly different significance results from other tests. DoEstRare is indeed based on a different hypothesis and could be used to explore new research insights, involving variant positions.

## Results

### Overview of DoEstRare

DoEstRare test aims to compare mutation position probability distributions on the region of interest (e.g. a gene) between cases and controls. If the distributions of rare variant positions are different in cases and controls, the gene is considered associated with the disease. This pattern could be expected when a specific domain of the protein is involved in the pathogenicity and causal mutations cluster in specific areas of the gene.

Simultaneously, we test a global burden hypothesis in DoEstRare to consider aggregated counts of mutations across the gene. Cases and controls may present equal mutation position distributions but different probabilities to present a rare mutation. This burden hypothesis consists in comparing average allele frequencies across the gene.

**Fig 1:**
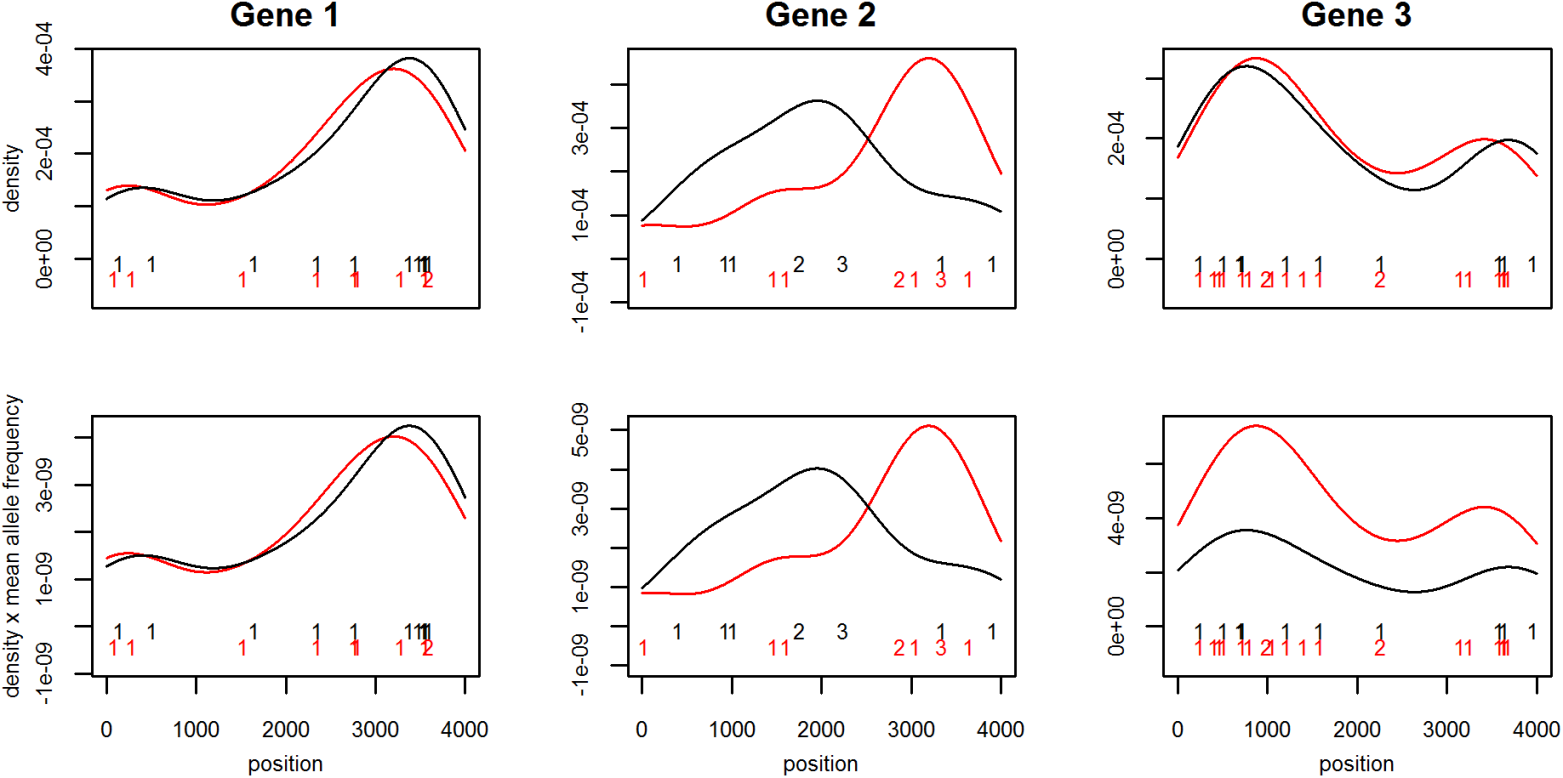
DoEstRare method illustration. The rare allele counts are represented on the gene in cases (red) and controls (black). From these counts are computed the density of mutation positions on the gene (top), and this density multiplied by the mean allele frequency (bottom), which is used by DoEstRare. A non-parametric method, the kernel density estimation using a Gaussian kernel, was used to estimate the mutation position density. Three genes have been simulated. Gene 1 presents the same mutation number (10) and the same mutation position distribution in cases and controls (no association with the disease). Gene 2 presents the same mutation number (10) but different mutation position distributions (association). Finally Gene 3 presents the same mutation position distribution but different mutation numbers (10 in controls and 20 in cases) (association).

The hypotheses of our test can be formulated as followed:

$$H_{0}\;:\;f^{A}=f^{U}\;and\;p^{A}=p^{U}$$

$$H_{1}\;:\;f^{A}\neq f^{U}\;or\;p^{A}\neq p^{U}$$

with *f*^*A*^ and *f*^*U*^, the mutation position density functions in affected (A) and unaffected (U) individuals; *p*^*A*^ and *p*^*U*^, the average allele frequencies. To illustrate these hypotheses, Gene 1 from **Fig 1** is not associated with the disease, as there is no difference in terms of position distribution and total mutation count. DoEstRare aims to identify situations like Gene 2 and Gene 3, where the mutation position distribution or the mutation number differs between cases and controls.

Further details about the construction of DoEstRare are described in the Methods section.

### Performance of DoEstRare

We conducted simulations to assess the type I error and the power of DoEstRare and 14 other association tests on rare variants, covering a large spectrum of existing strategies. The tests we compared are summarized in the **Table 1** with a categorization inspired by the review from Lee et al. (2014) [25].

**Table 1:** Rare variant association tests under comparison.

Positions	Category	Description of the strategy	Methods
No	Burden tests	Computation of a genetic score per individual corresponding to a binary variable.	CAST[4]
		Computation of a genetic score per individual corresponding to a weighted sum of genotypes.	WSS[6], VT[7], aSum[9]
	Variance-component tests	Test the variance of genetic effects.	C-alpha[10], SKAT[11], SKAT-O[12]
	P-value combination tests	Combination of p-values from single-marker tests.	ADA[14]
	Multi-genotype pattern	Analysis of multi-locus genotypes.	KBAC[8]
Yes	Sliding-window tests	A statistic is computed by genetic sliding window.	BOMP[18]
	Kernel matrix tests	A kernel matrix is used in the statistic to take into account physical distance between variants.	CLUSTER[20], KERNEL[19], PODKAT[21]
	Test on inter-marker distances	Physical distances between rare variants are computed. Weighted distance distribution functions are compared between cases and controls.	DBM[16]
	Rare variant density test	Comparison of rare variant position distributions and average allele frequencies on the gene, between cases and controls.	DoEstRare

Abbreviations: ADA, adaptive combination of P-values for rare variant association testing; aSum, data-adaptive sum test; BOMP, burden or mutation position; CAST, cohort allelic sum test; CLUSTER, test from Lin (2014); DBM, distance-based measure; KBAC, kernel-based adaptive cluster; KERNEL, test from Schaid et al. (2013); PODKAT, position-dependent kernel association test; SKAT, sequence kernel association test; VT, variable threshold; WSS, weighted sum statistic.

We simulated a 10kb gene with the backward coalescent model available in the COSI program [22], for a population of 10,000 haplotypes. We repeated the simulations to obtain 10,000 replicates of this haplotype set. The median number of simulated variants is 202 (2.5% and 97.5% quantiles: [172; 235]). The median number of variants with a MAF between 0.001 and 0.01 is 30 (2.5% and 97.5% quantiles: [18; 46]). From the population, we simulated the phenotype in order to obtain 1,000 cases and 1,000 controls.

#### Type I error analysis

**Fig 2:**
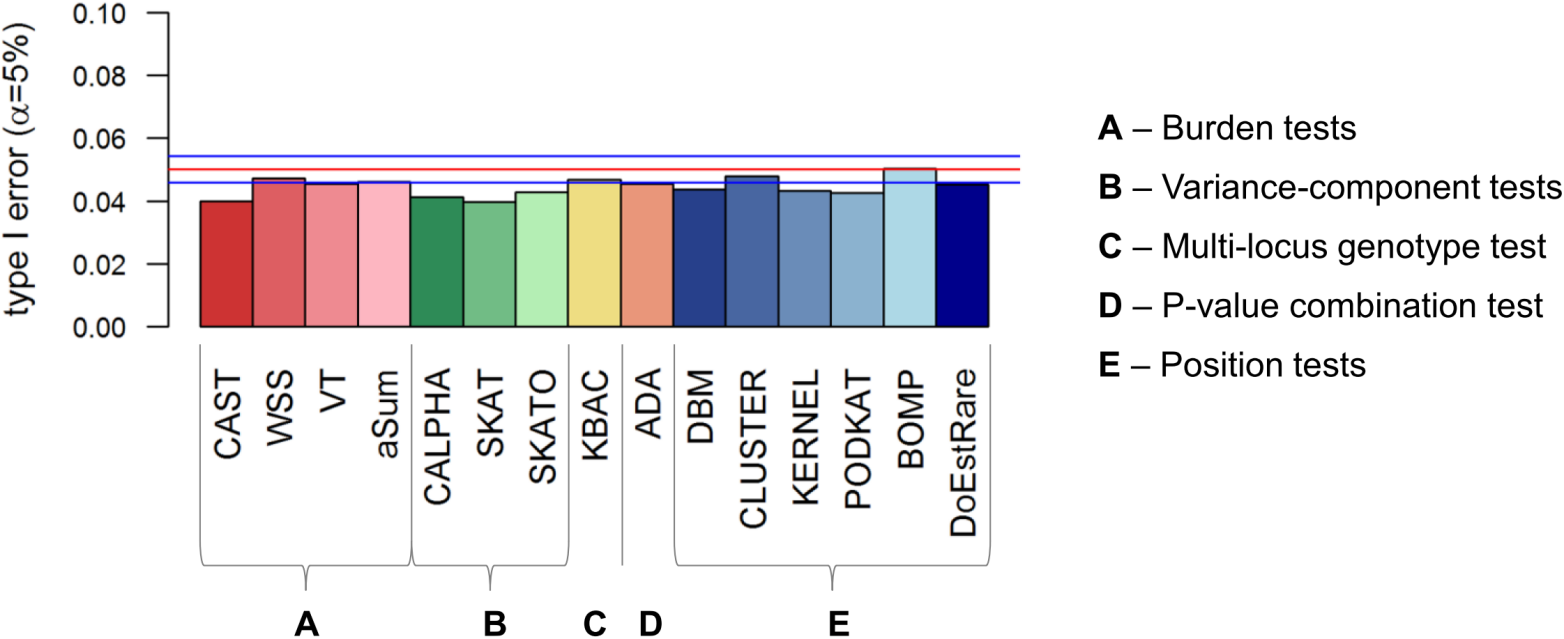
Type I error results at nominal level α = 5% based on 10,000 replicates. The red line corresponds to α = 5% and blue lines correspond to 95% confidence interval. Confidence interval is computed assuming that the number of false positives follows a binomial distribution with parameters 10,000 and 0.05.

We assessed type I errors for each test under comparison by simulating under the null hypothesis of no association between the gene and the disease status. Type I error results are based on 10,000 replicates and the **Fig 2** shows that all tests have correct type I error rates in terms of inflation of p-values [see S1A Table for type I error values]. Moreover some tests such as CAST and SKAT seem to be conservative. It has already been observed by Wu et al. (2011) that SKAT could be conservative with small sample sizes at low *α* levels [11].

#### Power analysis

**Fig 3:**
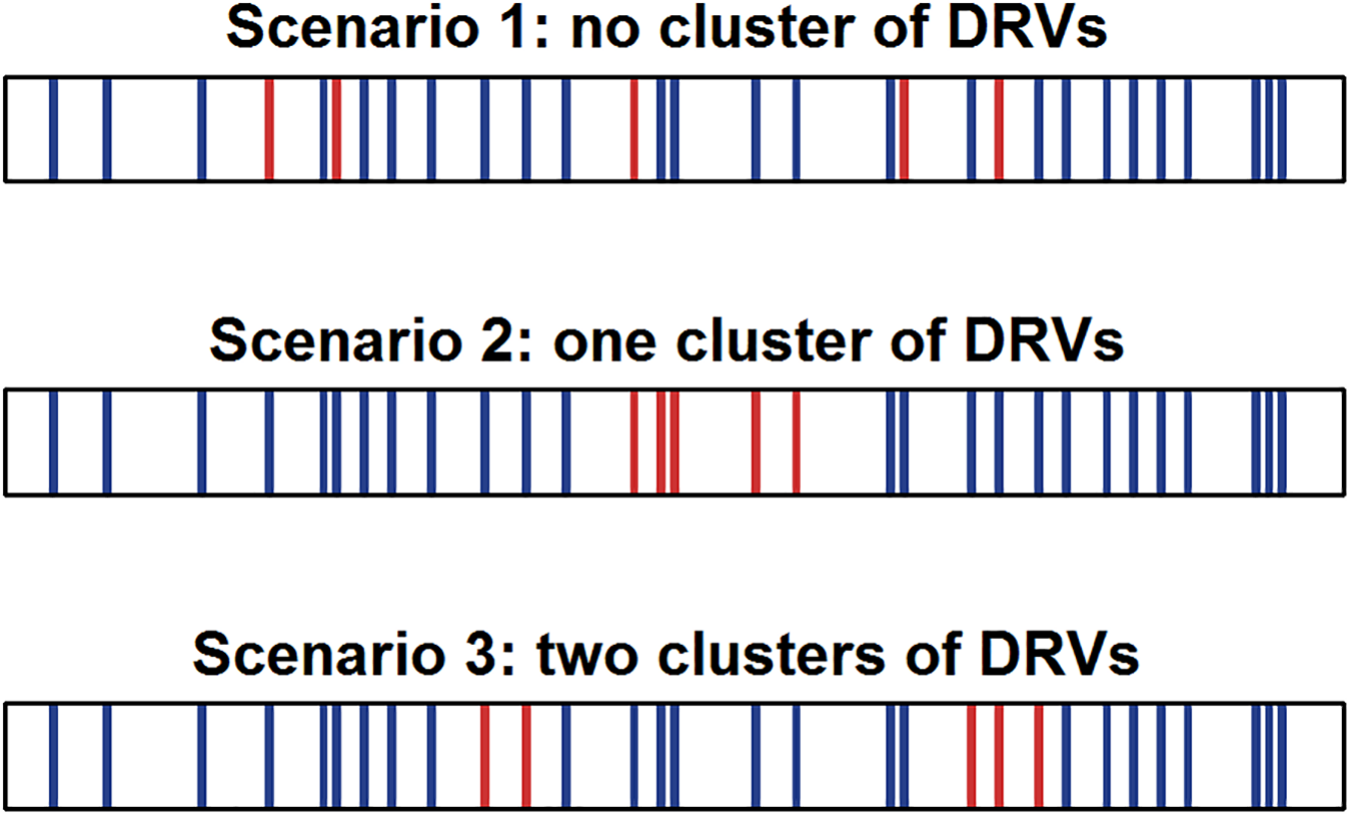
Simulation scenarios varying the DRV distribution. Each box represents a SNV on the gene. Blue boxes: non-causal variants. Red boxes: DRVs.

For the power analysis, we simulated a disease-associated gene under three main scenarios, varying the causal variant positions (**Fig 3**). In a first scenario, the causal variants are uniformly distributed on the gene. In the second and third scenarios, they are clustered in one or two specific areas of the gene. We also varied the proportion of causal variants between 5%, 10%, 15% and 20%.

**Fig 4:**
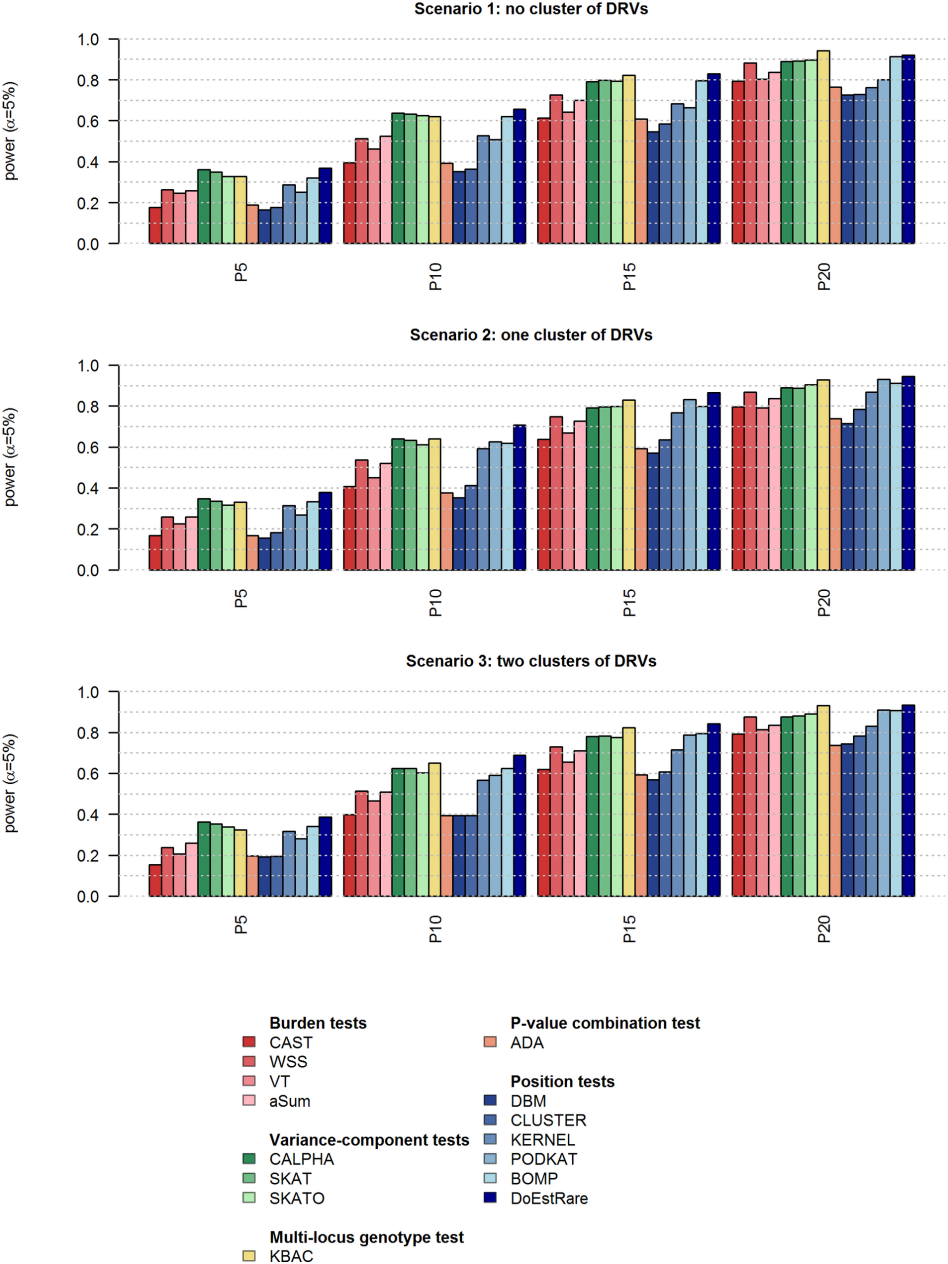
Power results at nominal level α = 5% based on 1,000 replicates. P5, P10, P15 and P20 correspond to 5%, 10%, 15% and 20% of DRVs in the gene. DRVs: disease-risk variants.

Focusing on scenario 1 results (**Fig 4**), in which DRVs are not clustered, an obvious observation is a power decrease with higher proportions of neutral variants [see S1B Table]. Nevertheless, in the context of no cluster of DRVs, some statistical tests seem to be more sensitive to the neutral variant inclusion than others. As it was observed with the comparison made by Basu and Pan (2011) [26], burden tests suffer from an important loss of power in a context of many non-causal variants, compared to variance-component tests. KBAC is also less noise-sensitive than burden tests, confirming that its statistic succeeds in better dissociating causal signals from noise. However ADA, which selects adaptively variants, is far less powerful than other tests in this context.

Regarding tests incorporating position information (“position tests”), DBM and CLUSTER tests perform badly with any proportion of DRVs. KERNEL and PODKAT tests are a bit more powerful than DBM and CLUSTER in this context of randomly distributed DRVs.

Finally BOMP and DoEstRare test perform well with any proportion of DRVs, compared to the other tests, with DoEstRare slightly better than BOMP, especially with low proportions of DRVs. DoEstRare is among the most powerful tests with variance-component tests and KBAC.

Focusing on scenarios 2 and 3, in which DRVs are clustered respectively in one and two areas, the tests that do not incorporate gene positions display obviously the same power as in scenario 1 [see S1C and S1D Table]. Tests incorporating position information such as CLUSTER, KERNEL and PODKAT and DoEstRare show a power increase in scenario 2, i.e. with one cluster of DRVs, for some proportions of DRVs [see S1A Fig for power comparison between scenarios]. However we can observe a power decrease with two small clusters compared to one large cluster. In these scenarios with clustered DRVs, variance-component tests and KBAC are still more powerful than a majority of position tests. Finally DoEstRare is the most powerful test in every simulated scenario from main scenarios 2 and 3.

### Application of DoEstRare to real data

We also applied our newly developed test and other association tests on rare variants to real data in order to study the significance similarities across the analyzed genes. We performed association tests on two pathologies, BrS and EOAD, in order to evaluate the stability of the comparison. Due to the number of tests under comparison, we used a multidimensional approach as Jeanmougin et al. (2010) [27]. We analyzed the significance similarities using a Principal Component Analysis (PCA) [28] on -log_10_(p-values) data.

#### BrS data

We applied 15 statistical tests (DoEstRare and 14 other tests) to BrS data. Sequence data for 163 candidate genes were available for 167 cases and 167 controls. Rare variants are defined as showing an MAF below 1%, and residing in coding DNA sequences (CDS) regions +/- 10 bp. We excluded from the PCA, all genes with missing p-values in at least one test. The reason of missing p-values is often due to the low number of variants in the gene. For some statistical tests such as DoEstRare and KERNEL, missing p-values are also due to the absence of rare mutations in cases or controls. We also excluded the DBM test, which returned more missing p-values than other tests. The PCA was performed on the remaining 58 genes (36% of the 163 genes in the targeted sequencing design) for the remaining 14 rare variant association tests. These genes represent a total of 1,462 rare variants, with a median of 15 rare variants per gene (min: 5; max: 441). In order to exhaustively compare BrS PCA results with EOAD PCA results, we set extra statistical tests (ADA, aSum, BOMP, CLUSTER, VT), which were performed only on BrS, as illustrative variables.

**Fig 5:**
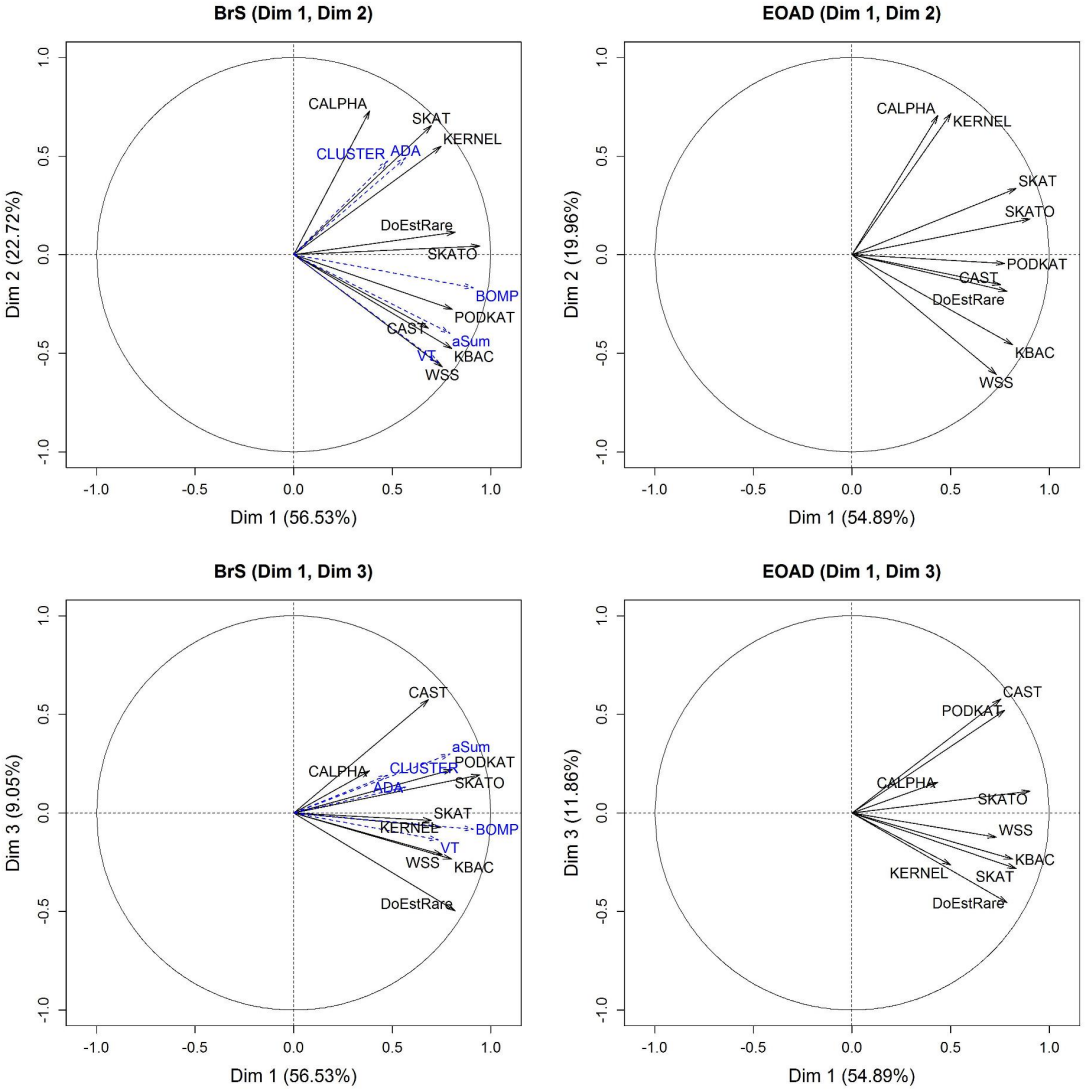
PCA correlation circle for BrS data (left) and EOAD data (right). PCA on -log_10_(p-value) is generated from the application of 9 association tests. BrS data includes 58 candidate genes. EOAD data includes 17409 autosomal protein coding genes. Illustrative variables are represented with blue dashed arrows.

The cumulative inertia explained by the first three axes of the PCA is about 88.31% of the total inertia. From the BrS correlation circle (**Fig 5**), all statistical tests are positively correlated to the first PC (56.53% of inertia). The second PC (22.72%) opposes C-alpha, SKAT and KERNEL tests (ADA and CLUSTER illustrative tests) on one side, and WSS and KBAC tests (aSum and VT illustrative tests) on the other side. A third PC which explains only 9.05% of the inertia opposes DoEstRare to CAST.

#### EOAD data

We applied DoEstRare and 8 rare variant association tests to the EOAD dataset, which contain whole exome sequences from 431 cases and 555 controls. Rare variants are still defined with an MAF inferior to 1% and a location in CDS regions. As for the previous analysis on BrS, we removed from the PCA, genes presenting a missing p-value for at least one test. We analyzed 17,409 autosomal protein-coding genes with 9 statistical tests. These genes represent a total of 273,390 rare variants, corresponding to a median number of variants per gene of 11 (min: 2; max: 901).

The first three axes explain 86.72% of the total inertia (**Fig 5**). As for BrS data, all statistical tests are positively correlated with the first PC (54.89% of inertia). The second PC (19.96%) opposes mainly C-alpha and KERNEL tests to WSS and KBAC tests. Finally the third PC (11.86%) opposes DoEstRare to CAST and PODKAT tests.

These axes summarize significance differences between the association tests. Focusing on DoEstRare, two association signals stand out by their significance: *KRTAP5-5* (transcript: ENST00000399676, p = 3.8e-07) and *CELA-3B* (transcript: ENST00000337107, p = 8e-06). *KRTAP5-5* is in the list of the 10 most significant association signals with only the tests SKAT (p = 3.8e-05) and SKAT-O (p = 6.4e-05). This gene is certainly a false positive gene as rare mutations in cases are clustered in a 3 bp region within a repetitive element and are carried by a few individuals. The second most associated gene, *CELA3B*, is far less significant for all the other tests, the minimum p-value obtained with SKAT (p = 2.6e-04). In this gene, rare variants present a different position distribution between cases and controls, and tend to cluster in a small genetic region in cases. On the contrary, the gene *NIPAL4* (transcript: ENST00000311946) with a high significance with most of the tests (CAST, WSS, SKAT-O, KBAC, PODKAT), is not well identified by DoEstRare (p = 1.13e-03). We can observe in this gene, a clear difference in the number of rare mutations between cases and controls, but not a clear difference of position distributions.

The significance levels of these three genes are shown in Manhattan plots [S2A–S2I Fig]. The localization of rare variants in cases and controls are represented in S3A–S3C Fig.

### Computation times

Computation times of the different statistical tests that are used in this paper are indicated in the **Table 2**. These times are based on the analysis a 10kb gene, simulated under the null hypothesis, and including 30 variants for 1000 cases and 1000 controls. For the standard permutation and bootstrap procedures, we did respectively 500 permutations or resampling.

**Table 2:** User CPU times for the different methods.

Test	Permutations/Bootstrap	Average time per gene (sec)	Total time (1000 genes)
CAST	No	0.013	0h 0min 13sec
WSS	Yes	26.221	7h 17min 1sec
VT	Yes	111.678	31h 1min 18sec
aSum	Yes	10.582	2h 56min 22sec
CALPHA	Yes	3.598	0h 59min 58sec
SKAT	No	0.091	0h 1min 31sec
SKAT	Yes (bootstrap)	1.326	0h 22min 6sec
SKAT-O	No	1.051	0h 17min 31sec
SKATO	Yes (bootstrap)	160.124	44h 28min 44sec
KBAC	Yes	0.187	0h 3min 7sec
ADA	Yes	25.318	7h 1min 58sec
DBM	Yes	7.933	2h 12min 13sec
CLUSTER	Yes	27.095	7h 31min 35sec
KERNEL	Yes	6.450	1h 47min 30sec
PODKAT	No	0.108	0h 1min 48sec
PODKAT	Yes (bootstrap)	1.459	0h 24min 19sec
BOMP	Yes	4.757	1h 19min 17sec
DoEstRare	Yes (standard)	22.617	6h 16min 57sec
DoEstRare	Yes (adaptive)	12.916	3h 35min 16sec

We note big differences in computation times between statistical tests. Of course these values depend highly on the implementation we used [see S1 Text for implementation details]. The most used statistical tests are CAST, SKAT and SKAT-O, as they are fast-running without a permutation or bootstrap procedure. DoEstRare computation time is quite high with a standard permutation procedure. This time can be greatly reduced with the adaptive permutation procedure we implemented and should be used in practice. The density estimation can be furthermore optimized in terms of computation time.

## Discussion

Here we propose a new association test for rare variants, called DoEstRare, to identify clusters of DRVs in genes. The DoEstRare strategy combines a “position test” and a burden test, so that it is still adapted to cases with randomly distributed DRVs. We also used, in the burden component, a weighting system to better discriminate risk variants from neutral variants.

First, we compared type I errors and powers, by conducting simulations under several genetic scenarios. These simulations scenarios were designed to assess statistical power in the context of high proportions of neutral variants. Simulations showed that DoEstRare is systematically the most powerful, alone or in conjunction with others for every scenario we simulated. DoEstRare performs well with or without clusters of DRVs. We also noticed by simulating scenarios varying proportion of DRVs, that DoEstRare is less noise-sensitive than burden tests.

In our power analysis, we also compared different strategies for testing rare variant association with a disease. We confirm that variance-component tests (C-alpha, SKAT, SKAT-O) and KBAC are better adapted to noise than burden tests and are powerful in every simulated scenario. There is no common benchmark in the literature for simulation designs to compare efficiently our results. However, Moutsianas et al. (2015) [29] compared different rare variant association tests and found that SKAT-O and KBAC have the highest mean power, across simulated scenarios varying sample sizes, effect locus sizes and neutral variant proportions, which is in accordance with our results. In our scenarios, SKAT-O, SKAT and C-alpha tests behave the same way because we simulated scenarios with high proportions of neutral variants. Compared to DoEstRare, these tests are still as powerful in most scenarios.

Most of the existing tests incorporating position information, except PODKAT and BOMP, are less powerful than variance-component tests and KBAC, in every scenario. KERNEL, DBM and PODKAT are more powerful in the presence of a cluster of DRVs, confirming the use of position information in their statistic. We also noted some power differences between scenarios with one and two clusters of DRVS. Indeed some tests are significantly less powerful in the scenario with two clusters. This observation may be related to cluster sizes being smaller in this scenario, for the same proportion of DRVs.

Simulation results depend on many factors due to the complexity of a group of rare variants. In our simulated scenarios we varied the proportion of causal variants in the gene and also the number of clusters of DRVs. Currently, there is still poor knowledge about the underlying biological mechanisms, likely to differ greatly between diseases. That is why it is hard to assess the realism of these two parameters. We used constant ORs for disease risk variant effects while it is unlikely that all causal variants in a gene have the same effect. Some authors suppose very rare variants to present stronger effects and set the regression coefficients of the logistic model as a decreasing function of the MAF [11,12]. Moreover, every simulated gene is a 10kb region while the real genetic structure is more complicated with very heterogeneous gene sizes and variant numbers.

We could assess the power of our statistical test DoEstRare and usual tests, with the analysis of simulated data. However, to test their behavior on biological datasets, we have applied up to 14 association tests, in addition to DoEstRare, on rare variants to BrS and EOAD data and investigated the significance similarities between the results from the different tests. This comparison may help in choosing the best test combination when designing a rare variants project and in interpreting differential test results. Both BrS and EOAD PCA representations underlined the same tendencies. All statistical tests are correlated with the first principal component, which means that significance results provided by different tests show globally the same tendency. The second source of inertia in significance results is due to some statistical tests giving partially different results. This may be related to the underlying genetic structure behind the disease. The main significance differences, according to the second principal component, are between the group including C-alpha and KERNEL, and the group including WSS and KBAC. The third component, which explains a low part of the inertia, opposes DoEstRare to CAST. Finally SKAT-O seems to be a good compromise as it provides similar results than other tests. We could observe some differences in the correlation structure when analyzing BrS data and EOAD data. For instance, the correlation of PODKAT and SKAT significance results with the other tests differs between BrS and EOAD datasets. One explanation is that correlation structure between statistical results may depend on the studied pathology, as statistical tests assume different biological models. Interestingly, although we observe some correlations between association tests based on similar hypotheses, we do not observe a clear categorization. This issue should be further investigated with the study of other diseases.

With the application of rare variant association tests to real data, we highlighted several practical issues. In this article, we considered analyzing all rare variants without prior functional information. Criteria to incorporate only the most potential disease-risk rare variants in the analysis are not well defined in the literature, as the genetic architecture is specific of the studied disease. It is possible to take into account only those variants that have been previously functionally annotated [25,30,31], for example with sequence ontology terms describing their impact on the coded protein, in the context of gene analysis. Regarding the tests that incorporate position information, the delineation of the analyzed region is even more important as positions are relative to the bounds. It should be added that using tests to detect clusters of DRVs is irrelevant when the analyzed gene contains very few variants. The observed number of rare variants per gene depends greatly on the sample population size of the study. Indeed by screening more individuals in a study, it is more probable to identify very rare mutations. The sampling design is also an important factor in association analyses, as Nicolas et al. (2015) [24] identified an association signal when applying rare variant association test to EOAD patients with a positive family history. In this article we chose to analyze all 431 EOAD patients, whose 185 patients present a family history.

When applying statistical tests to real data, the use of an adaptive permutation procedure [32] is needed to reduce computational times and we implemented it for most of tests. Even using this strategy, encountering a high association signal may be time-consuming compared to association tests using an approximate statistic distribution under the null hypothesis. Moreover, taking into account position information is very useful to discover clusters of DRVs, but the delineation of the analyzed region is a supplementary step in the analysis workflow requiring reasoning. For the analysis of BrS data, we chose to use the definition of captured coding sequences. For the EOAD data, as capture designs were differing among patients, we used CDS annotations. Of course, by analyzing CDS regions, we may have missed few important variants situated in splice regions. The presence of clustered rare variants is, in some cases, due to technical artefacts as some regions are difficult to sequence. In EOAD results, a gene presents a very high significance with DoEstRare but is a false positive due to a cluster of rare mutations in a very small region for a very few cases. DoEstRare is also interesting to explore these short genomic regions with a low sequencing quality.

The relevance of a test depends highly on the underlying biological structure when assessing the association between a group of rare genetic variants and a disease. Each statistical test for rare variants is based on relative complex assumptions aiming to translate mathematically the disease mechanisms. We recommend, in order to identify the maximum association signals, to perform different statistical tests covering various strategies.

DoEstRare enables to incorporate position information and is powerful to detect clusters of disease risk variants. DoEstRare is a good alternative test to use in addition to classical strategies in order to explore genetic architectures involving functional domains. This test is advantageous in the context of analyzing long transcripts which are susceptible to contain important sub-regions. However, the density estimation of rare variant positions may be limited by testing a small group of variants. As discussed previously, DoEstRare is sensitive to short genetic areas that are not well sequenced, which may result in the presence of false positives. The quality control is an important step in the analysis of rare variants, and DoEstRare can also be used to identify problematic sequences.

Our newly developed test DoEstRare is so far designed for deleterious rare alleles in case/ control studies. In this article we did not take into account genetic population structure in rare variant association tests. However it has already been shown that this could impact significance results [33,34]. It can be developed in four directions: (i) adapting weights in the DoEstRare statistic in order to consider a mixture of protective and deleterious variants, or incorporating functional information; (ii) applying DoEstRare to quantitative traits by using a latent binary status, whose distribution probability depends on the phenotype distribution; (iii) exploring the choice of the kernel used in the density function estimations to reduce computational times; (iv) incorporating population stratification components in the computation of mean allele frequencies.

## Methods

### Notations

Let **X** be the matrix of genotypes with *X*_*ij*_ the count of rare alleles for the *i*-th individual and *j*-th rare variant, varying between 0, 1 or 2 rare alleles. Let ***Y*** be the vector of phenotypes with *Y*_*i*_ = 1 if the *i*-th individual is a case, *Y*_*i*_ = 0 if else. Let *l*_*j*_ be the position of the *j*-th rare variant. The number of affected (A) and unaffected (U) individuals are respectively *N*^*A*^ and *N*^*U*^, with *N* the total number of individuals. The number of rare variants in the gene is *P*.

### Tests under comparison

A first category of rare variant association tests is called burden tests [4,6,7,9], and consists in summarizing (or collapsing) the genetic information across the variants or across the individuals into a single value. A simple approach consists in computing for each individual a genetic score corresponding to a weighted sum of minor allele counts

$$S_{i}=\sum_{j=1}^{P}w_{j}X_{ij}$$

with *w*_*j*_ the weight for variant *j*. For example, the WSS test accords a more important weight to rare variants, as they may be more likely to have an effect on disease susceptibility. Weights differ between approaches according to biological assumptions. Finally the association between the genetic score and the disease status is tested.

Another category are the variance-component tests [10,11]. It has been developed to deal with the presence of opposite effects (deleterious and protective variants) and difference of effect magnitude (moderate effect to no effect). They test unusual variance of genetic effects on disease susceptibility in a group of variants. SKATs enable also more complex disease susceptibility models than the linear logistic regression model.

Because the proportion of deleterious and protective rare alleles is not known, some statistical tests combine both a burden test and a variance-component test such as SKAT-O [12] in order to preserve highest power. Indeed it has been observed by Basu et al. (2011) [26] that burden tests perform best when the gene includes a large amount of causal variants with the same effect whereas variant-component tests perform best in situations with protective or a lot a non-causal variants. Combined tests are developed to take advantage of the both strategies. To simplify our classification, we put SKAT-O into the variance-component test category.

Another strategy in association tests is to combine p-values obtained by single-marker tests that are commonly used in GWASs such as the ADA test [14].

The KBAC test [8] is a test considering multi-locus genotypes, i.e. the combinations of alleles across the genetic region of interest. This strategy aims to account for potential within-gene or between-gene interactions. It also uses a weighting system to better account for potential risk variants.

Finally several tests incorporate physical positions of rare variants [16–21]. A simple approach to detect a cluster of DRVs in a gene is to use a sliding-window approach. The genetic region of interest is divided into windows and a rare variant association test is performed for each window. Because the size and the location of the cluster are usually unknown, sliding windows of different sizes are commonly considered. This strategy is used in the BOMP test, proposed by Chen et al. (2013), and the test developed by Ionita-Laza et al. (2012). We didn’t use the scan test from Ionita-Laza et al. (2012) as it is more suitable for large regions. Other tests such as KERNEL, CLUSTER and PODKAT, incorporate position information in a kernel matrix that measures distances between pairs of rare variants. In this article we propose a rare variant association test, DoEstRare, which is a combination of a burden test and a test comparing mutation position distributions.

Tests are described with more details in supplementary methods [see S1 Text].

### DoEstRare test

#### Computation of the test statistic

DoEstRare aims to compare both the rare variant position distributions and allele frequencies between cases and controls. To test these two aspects, the statistic computes the area between the two mutation position density curves, each multiplied by the corresponding mean allele frequency, computed across all rare variants:

$$STAT=\int_{1}^{Lg}|\hat{p^{A}}\times {\hat{f}}_{}^{A}(pos)-\hat{p^{U}}{\hat{f}}_{}^{U}(pos)|dpos$$

with *Lg* denotes the length of the gene in bp. $\hat{p^{A}}$, $\hat{p^{U}}$, ${\hat{f}}^{A}$ and ${\hat{f}}^{U}$ are estimators for respectively mean allele frequencies and position density functions whose computation will be explained in next sections. Without the burden components $\hat{p^{A}}$ and $\hat{p^{U}}$, the statistic is similar to the total variation distance, used to compute a distance between the two probability density functions ${\hat{f}}^{A}$ and ${\hat{f}}^{U}$ [35].

#### Estimation of density functions

Position density functions *f*^*A*^ and *f*^*U*^ are estimated using a non-parametric way with the Gaussian kernel density estimation [36].

$${\hat{f}}^{A}(pos)=\frac{1}{bw}\sum_{j=1}^{P}\;w_{j,density}^{A}\times K(\frac{pos-l_{j}}{bw})\;and\;{\hat{f}}^{U}(pos)=\frac{1}{bw}\sum_{j=1}^{P}w_{j,density}^{U}\times K(\frac{pos-l_{j}}{bw})$$

with *bw* the bandwidth (smoothing parameter) and *K*(.), the Gaussian kernel.

$w_{j,density}^{A}$ and $w_{j,density}^{U}$ are ratios of mutations at the *lj*-th position in cases and controls.

$$w_{j,density}^{A}=\frac{m_{j}^{A}}{\sum_{j=1}^{P}m_{j}^{A}}$$

$$w_{j,density}^{U}=\frac{m_{j}^{U}}{\sum_{j=1}^{P}m_{j}^{U}}$$

with $m_{j}^{A}=\sum_{i=1}^{N^{A}}X_{ij}$ and $m_{j}^{U}=\sum_{i=1}^{N^{U}}X_{ij}$ the observed counts of mutations for the *j*-th variant in cases and controls.

#### Burden components

To test the burden hypothesis, we estimate a weighted allele frequency average in cases and controls. The weight system enables to better discriminate high potential causal variants from neutral variants. The burden component expressions are:

$$\hat{p^{A}}=\frac{1}{P}\sum_{j=1}^{P}\frac{w_{j}}{\sum_{j=1}^{P}w_{j}}\frac{m_{j}^{A}}{{2N}^{A}}\;\hat{p^{U}}=\frac{1}{P}\sum_{j=1}^{P}\frac{w_{j}}{\sum_{j=1}^{P}w_{j}}\frac{m_{j}^{U}}{{2N}^{U}}$$

with *w*_*j*_ the weight for the *j*-th variant.

Under the assumption that all causal variants are deleterious, (i.e. variants that are enriched in cases present a more important weight), we assume that the count $M_{j}^{A}$ of rare mutations in cases for a variant *j* follows, under the null hypothesis, a binomial distribution $B(2N^{A},\hat{q_{j}^{U}})$ with $\hat{q_{j}^{U}}$ the estimate of the minor allele frequency in controls:

$$\hat{q_{j}^{U}}=\frac{m_{j}^{U}+1}{2N^{U}+2}$$

The weight *w*_*j*_ is defined as the probability to present less than the observed count $m_{j}^{A}$.

$$w_{j}=P(M_{j}^{A}\leq m_{j}^{A})=\sum_{k=0}^{m_{j}^{A}}(\begin{array}{c} 2N^{A}\\ k \end{array}){\left(q_{j}^{U}\right)}^{k}{\left(1-q_{j}^{U}\right)}^{2N^{A}-k}$$

#### Significance

The significance of the test is evaluated with a standard phenotype permutation procedure. For each permutation *b* ∈ {1,…,*B*}, the phenotypes labels are randomly shuffled (permuted) and the statistic *STAT*^(*b*)^ is calculated. As the statistic is an area, which means a positive real number, the p-value is defined, in the context of standard phenotype permutation procedure, by $\frac{\sum_{b=1}^{B}(STAT^{\left(b\right)}\geq STAT)+1}{B+1}$ [37], with *B* the total number of permutations. An adaptive permutation procedure can also be used to reduce computational times, in the context of large data [32].

### Simulation framework

**Fig 6:**
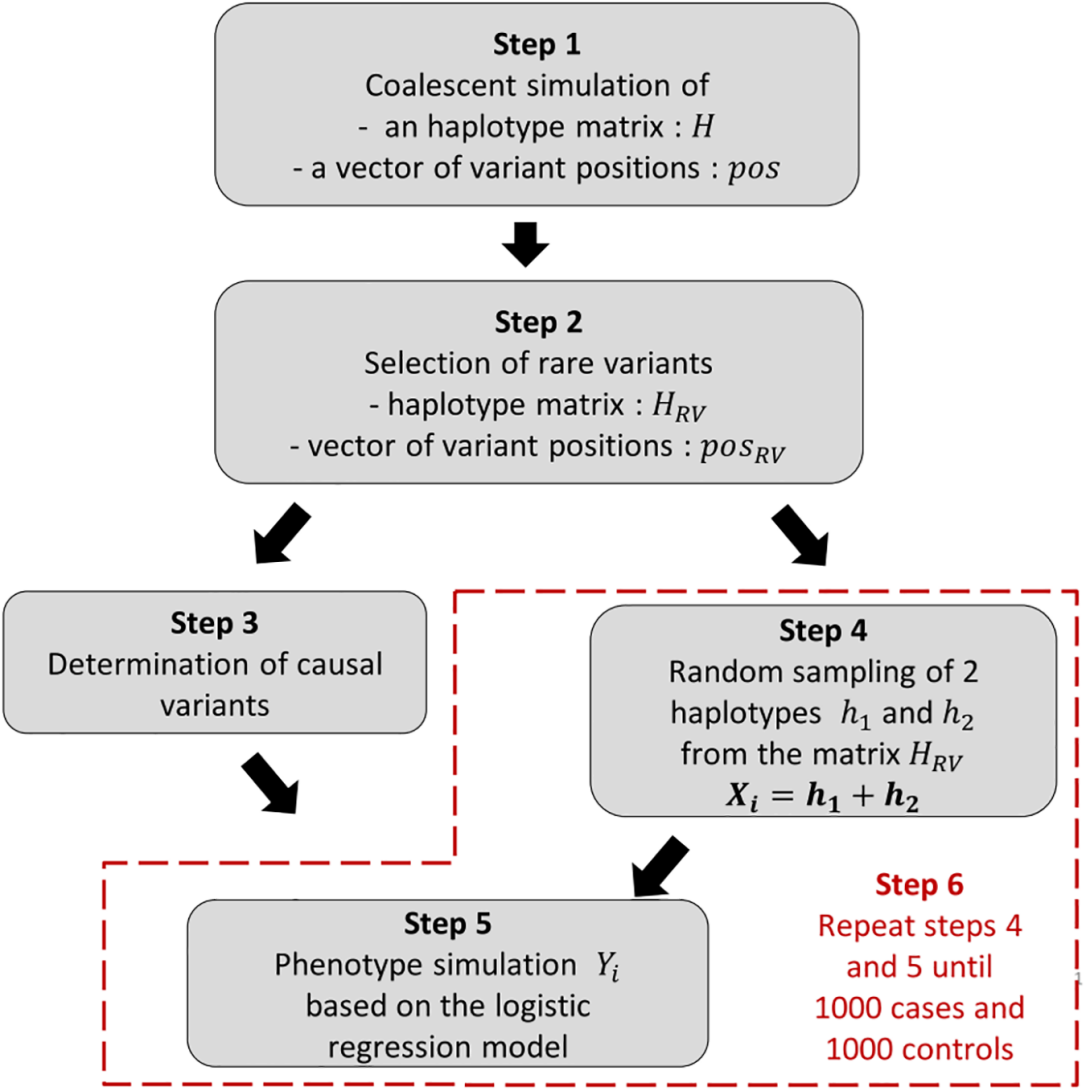
Simulation workflow. The different steps of the simulation workflow are further detailed in the article. The red dashed frame represents the step 6 which consists in the repetition of steps 4 and 5.

We conducted genetic simulation studies to evaluate and compare the performance of DoEstRare in terms of power and type I error. Our simulation workflow for each replicate is described with the following steps. It briefly consists in generating a haplotype matrix from which are sampled cases and controls, the disease risk model being a logistic regression model (see **Fig 6**).

Step 1: We generate 10,000 haplotypes for a 10kb region using a backward coalescent model implemented in the COSI program [22]. Parameters correspond to what is called the “bestfit” model by Schaffner et al. (2005), which were obtained by calibration. Haplotypes are sampled from what corresponds to the European population in this model.

Step 2: In the haplotype matrix generated in step 1, we select rare variants with a MAF ∈ [0.001;0.01]. We set a minimal MAF to avoid a lot of non-observed causal mutations in the simulated data, a framework leading to the null hypothesis model because of the very low frequency of a large proportion of variants.

Step 3: We determine rare causal variants for the logistic regression model. Causal variants are determined according different scenarios related to their positions on the gene. These scenarios are explained further in the simulation framework description.

Step 4: We sample two haplotypes from the haplotype matrix generated in step 1 to constitute the genotype data *X*_*i*_ for the *i*-th individual.

Step 5: The phenotype of the *i*-th individual is simulated with the following logistic regression model:

$$logit(P(Y_{i}=1|X_{i}))=\beta_{0}+{X_{i}}^{T}\beta$$

with *β*_0_ the intercept and ***β*** the vector of regression coefficients for the genetic effects. We set $\beta_{0}=\log\left(\frac{0,05}{1-0,05}\right)$ so that 5% of individuals without any rare mutation are affected. In the context of rare variants, this value is close to the disease prevalence. For regression coefficients, we chose *β*_*j*_ = log(OR_*j*_) with *OR*_*j*_ = 3 if the *j*-th is a causal variant, else *β*_*j*_ = 0. The disease status of the individual *i* is sampled according to the Bernoulli distribution of probability *P*(*Y*_*i*_ = 1|*X*_*i*_).

Step 6: We repeat steps 4 and 5 until we obtain 1,000 cases and 1,000 controls.

Three main scenarios (**Fig 3**) are considered in relation to the positions of causal variants. In a first scenario, DRVs are not clustered in any specific area and are randomly sampled without replacement on the whole gene. In the second and third scenarios, DRVs cluster respectively in one and two areas. In these scenarios, initial causal variant positions correspond to the median of all variant positions for the second scenario, and to the quantiles 1/3 and 2/3 for the third scenario. Then DRVs are extended from initial causal positions to the neighbor variants until the specified number of DRVs is reached. We set the number of DRVs so that the proportions vary between 5%, 10%, 15% and 20% of the total variants within a gene (noted scenarios P5, P10, P15 and P20). In the context of clustered DRVs, these proportions are related to cluster window sizes. Indeed, cluster window sizes are larger with higher proportions of DRVs.

The performances of the different tests (see section Tests under comparison) are compared in terms of power and type I error. We defined the type I error and the power as

$$\frac{\sum_{r=1}^{R}I(p-value_{r}\leq \alpha )}{R}=\left\{ \begin{array}{cc} type\;I\;error(\alpha ) & if\;H_{0}\;situation\\ power(\alpha ) & if\;H_{1}situation \end{array}\right.$$

with *r* ∈ {1,…,*R*} the replicate index. We did 1,000 replicates for each scenario of the power analysis and 10,000 replicates for type I error analysis. Power and type I errors were computed for the test significance level at α = 5%.

We applied DoEstRare and 14 other rare variant association tests on simulated data. We set B = 500 permutations for each permutation-based test, i.e. all tests except CAST, SKAT, SKAT-O and PODKAT.

### Real data

We performed DoEstRare and other rare variant association tests (see section Tests under comparison) on BrS and EOAD data. We analyzed the significance result similarities between the different tests by using a PCA [28]. The PCA data is the matrix containing (−*log*_10_(*p*−*value*))_*jt*_ for gene *j* in row and statistical test *t* in column. The PCA was performed with the R package FactoMineR [38].

#### Data from the BrS study

We applied DoEstRare and 14 rare variant association tests to BrS data published by Le Scouarnec et al. (2015) [23]. In this study, rare variant association tests were conducted to identify new genes of susceptibility for BrS. A significant enrichment of *SCN5A* rare variant carriers was observed in BrS patients.

In this study, cases include 167 patients diagnosed with BrS, and controls include 167 individuals aged over 65-year old and showing no history of cardiac arrhythmia. Both cases and controls are individuals of European origin.

This is a candidate gene study in which coding sequences of 163 candidate genes have been captured and sequenced. In this publication, burden test results for 45 genes are published. These genes have been previously shown to be related to cardiac arrhythmias or conduction defects and/or sudden cardiac death. The functional units tested are genes and more specifically the coding regions with a margin of 10 bp to take into account splicing sites.

In the present study, rare genetic variants were defined as variants with a MAF<1% in the Exome Aggregation Consortium (ExAC) database for the Non-Finnish European population (NFE) (release 0.3 downloaded at ftp://ftp.broadinstitute.org/pub/ExAC_release/release0.3/ExAC.r0.3.sites.vep.vcf.gz) [39]. To avoid false positives, we excluded variants found in more than 5% of cases or controls but absent from ExAC database. Unlike the burden test framework described in the original publication, we analyzed all variants regardless their Sequence Ontology (SO) terms, estimated with Ensembl (http://www.ensembl.org), which describe the type of consequence of the mutation.

We evaluated significance of association tests using a standard permutation procedure (excluding CAST, SKAT, SKAT-O and PODKAT) with 1,000 phenotype permutations for all genes except *SCN5A*, which is a major gene implicated in BrS and where we performed 200,000 permutations.

Note: More details about population sampling, sequencing and variant calling can be found in the publication of Le Scouarnec et al. (2015) [23].

#### Data from the Alzheimer study

We performed rare variant association tests on EOAD data from Nicolas et al. (2015) [24]. In this study, a significant enrichment of SORL1 rare variants was detected in EOAD patients.

Published data, after quality control preprocessing, include 498 controls from 5 different French cities and 484 EOAD patients recruited by the French National CNR-MAJ consortium (205 patients with positive family history). We brought some modifications to the initial design in order to allow a robust comparison between tests. First, we decided to minimize technical biases by including only cases and controls that were sequenced by either Agilent SureSelect Human All Exons V5 or Agilent SureSelect Human All Exons V5UTR capture designs. Additional controls from another French city were also included, due to the French Exome project’s progress. Our analysis finally compared 555 controls to 431 EOAD patients.

In this exome study, we annotated variants with Variant Effect Predictor (Ensembl). For each protein coding gene, we analyzed the “canonical” transcript: the transcript which presents (1) the longest CDS length, (2) if CDS lengths are equal, the longest transcript length with UTR regions. A total of 19,076 autosomal protein coding genes were annotated. For association tests incorporating position in the transcript, we used CDS regions and each variant was annotated with a CDS position.

Filters on genetic variant are the same as for the BrS data analysis. Rare genetic variants present a MAF<1% in the ExAC database for the NFE population and present a MAF in cases and in controls both less than 5%. To avoid false positives we excluded all rare variants which were very significant, as they could influence results from gene-based tests. These variants need to be checked apart from the analysis. Variants with a p-value less than 1e-04 by single-marker test (Fisher exact) were removed.

Due to the data size, we used the adaptive permutation procedure described by Che et al. (2014) to reduce computational times [32]. We set, as parameters, the adjusted nominal significance level to *α* = 5e-07, with a precision of *c* = 0.2. We chose to not apply all statistical tests and selected several tests per category. We applied CAST and WSS as burden tests, SKAT and SKAT-O as variance component tests, KBAC as a multi-locus genotype test, KERNEL, PODKAT and DoEstRare as position tests. Some statistical tests as ADA and CLUSTER were not easily adaptable for adaptive permutation and thus were not included in this round of analyses.

## Supporting information

S1 Table Power and type I error tables. Values of type I errors and powers assessed with the analysis of simulated data.
(PDF)

S1 Fig Power comparison between simulated scenarios. The Fig A is another illustration of power results to better compare simulated scenarios, represented in Fig 4.
(PDF)

S2 Fig Manhattan plots for EOAD results. From Fig A to Fig I, are represented significance results for the 17,409 autosomal genes that were analyzed. Only the names of the three genes, *KRTAP5-5*, *CELA3B* and *NIPAL4*, are indicated. The red line corresponds to a significance level of 2.5e-06 (5% adjusted with a Bonferroni correction for 20,000 genes).
(PDF)

S3 Fig Mutation position density plots for EOAD results. From Fig A to Fig C, are represented allele counts and mutation position densities for the three genes, *KRTAP5-5*, *CELA3B* and *NIPAL4*, in cases and controls. Density functions for mutation positions were estimated with a Gaussian kernel.
(PDF)

S1 Text Supplementary methods. Further details about the rare variant association tests we compared.
(PDF)
