# Supplementary material for: DoEstRare: A statistical test to identify local enrichments in rare genomic variants associated with disease
Source: PLoS One. 2017 Jul 24;12(7):e0179364. doi: 10.1371/journal.pone.0179364 (PMC5524342; doi:10.1371/journal.pone.0179364)
Supplement: S1 Table — Values of type I errors and powers assessed with the analysis of simulated data. (PDF) [file pone.0179364.s001.pdf]

S1 Table: Power and type I error tables

Table A. Type I error results at nominal level α=5% based on 10,000 replicates.

	Type I error
**CAST**	0.0399
**WSS**	0.0472
**VT**	0.0455
**aSum**	0.0461
**C-alpha**	0.0412
**SKAT**	0.0398
**SKAT-O**	0.0428
**KBAC**	0.0467
**ADA**	0.0455
**DBM**	0.0436
**CLUSTER**	0.048
**KERNEL**	0.0433
**PODKAT**	0.0426
**BOMP**	0.0504
**DoEstRare**	0.0452

Table B. Power results for the scenario 1 at nominal level α=5% based on 1000 replicates.

	**P5**	**P10**	**P15**	**P20**
**CAST**	0.176	0.395	0.612	0.794
**WSS**	0.262	0.511	0.726	0.881
**VT**	0.245	0.462	0.642	0.802
**aSum**	0.258	0.524	0.699	0.835
**C-alpha**	0.36	0.636	0.791	0.888
**SKAT**	0.35	0.632	0.798	0.892
**SKAT-O**	0.328	0.625	0.793	0.895
**KBAC**	0.328	0.62	0.822	0.941
**ADA**	0.189	0.391	0.607	0.763
**DBM**	0.165	0.352	0.546	0.726
**CLUSTER**	0.177	0.363	0.583	0.728
**KERNEL**	0.286	0.526	0.682	0.761
**PODKAT**	0.25	0.508	0.663	0.8
**BOMP**	0.321	0.62	0.795	0.914
**DoEstRare**	0.369	0.657	0.828	0.92

P5, P10, P15 and P20 correspond to 5%, 10%, 15% and 20% of DRVs in the gene. DRVs: disease-risk variants.

Table C. Power results for the scenario 2 at nominal level α=5% based on 1000 replicates.

	P5	P10	P15	P20
**CAST**	0.166	0.408	0.638	0.796
**WSS**	0.259	0.537	0.748	0.868
**VT**	0.224	0.451	0.668	0.791
**aSum**	0.258	0.521	0.726	0.836
**C-alpha**	0.348	0.639	0.792	0.89
**SKAT**	0.335	0.633	0.797	0.887
**SKAT-O**	0.317	0.612	0.799	0.904
**KBAC**	0.33	0.64	0.83	0.927
**ADA**	0.167	0.375	0.592	0.739
**DBM**	0.155	0.353	0.57	0.715
**CLUSTER**	0.181	0.413	0.635	0.783
**KERNEL**	0.313	0.592	0.768	0.868
**PODKAT**	0.267	0.626	0.831	0.931
**BOMP**	0.333	0.619	0.798	0.912
**DoEstRare**	0.378	0.707	0.865	0.944

P5, P10, P15 and P20 correspond to 5%, 10%, 15% and 20% of DRVs in the gene. DRVs: disease-risk variants.

Table D. Power results for the scenario 3 at nominal level α=5% based on 1000 replicates.

	P5	P10	P15	P20
**CAST**	0.154	0.399	0.619	0.791
**WSS**	0.237	0.514	0.729	0.876
**VT**	0.207	0.466	0.655	0.814
**aSum**	0.26	0.509	0.709	0.835
**C-alpha**	0.361	0.623	0.78	0.876
**SKAT**	0.352	0.623	0.782	0.881
**SKAT-O**	0.339	0.601	0.775	0.89
**KBAC**	0.324	0.65	0.823	0.931
**ADA**	0.196	0.394	0.593	0.736
**DBM**	0.192	0.394	0.569	0.743
**CLUSTER**	0.195	0.393	0.606	0.782
**KERNEL**	0.317	0.566	0.714	0.83
**PODKAT**	0.28	0.591	0.787	0.909
**BOMP**	0.341	0.624	0.795	0.906
**DoEstRare**	0.387	0.689	0.841	0.933

P5, P10, P15 and P20 correspond to 5%, 10%, 15% and 20% of DRVs in the gene. DRVs: disease-risk variants.
