# Supplementary material for: DoEstRare: A statistical test to identify local enrichments in rare genomic variants associated with disease
Source: PLoS One. 2017 Jul 24;12(7):e0179364. doi: 10.1371/journal.pone.0179364 (PMC5524342; doi:10.1371/journal.pone.0179364)
Supplement: S1 Fig — The Fig A is another illustration of power results to better compare simulated scenarios, represented in Fig 4. (PDF) [file pone.0179364.s002.pdf]

S1 FIG: POWER COMPARISON BETWEEN SIMULATED SCENARIOS

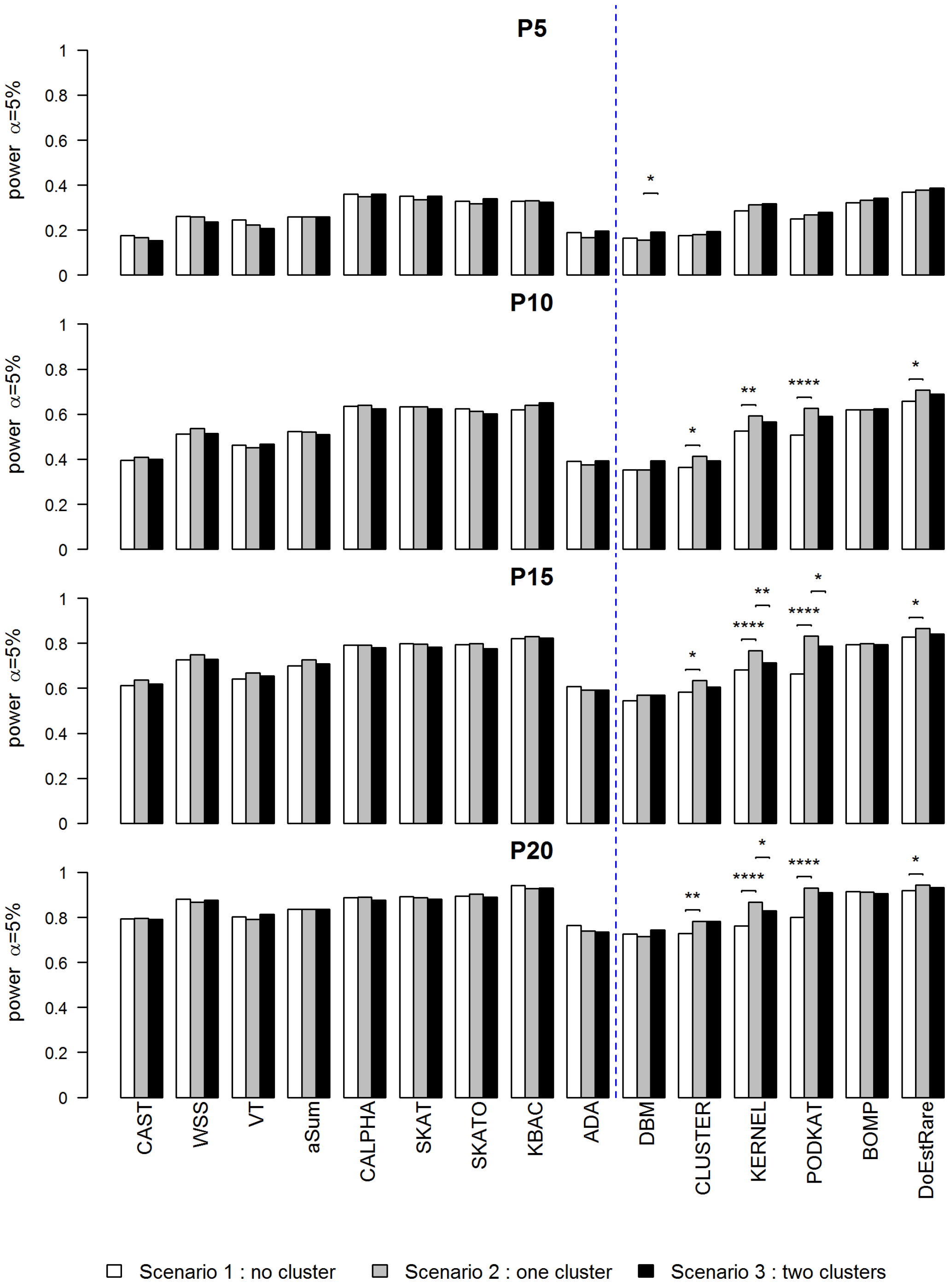

Fig A. Power comparison between scenarios. A Fisher exact test has been performed to test the independency between the gene significance at level α=5% and the compared scenarios [see S1 Text, p15]. We compared scenarios 1 and 2 (resp. 2 and 3). ns: p>0.05, *: p≤0.05, **: p≤0.01, ***: p≤0.001, ****: p≤0.0001. The dashed blue line separates "position tests" (DBM, CLUSTER, KERNEL, PODKAT, BOMP and DoEstRare) from other tests.
