# Supplementary material for: DoEstRare: A statistical test to identify local enrichments in rare genomic variants associated with disease
Source: PLoS One. 2017 Jul 24;12(7):e0179364. doi: 10.1371/journal.pone.0179364 (PMC5524342; doi:10.1371/journal.pone.0179364)
Supplement: S2 Fig — From Fig A to Fig I, are represented significance results for the 17,409 autosomal genes that were analyzed. Only the names of the three genes, KRTAP5-5, CELA3B and NIPAL4, are indicated. The red line corresponds to a significance level of 2.5e-06 (5% adjusted with a Bonferroni correction for 20,000 genes). (PDF) [file pone.0179364.s003.pdf]

S2 Fig: Manhattan plots for EOAD results

From Fig A to Fig I, are represented significance results for the 17,409 autosomal genes that were analyzed. Only the names of the three genes, *KRTAP5-5*, *CELA3B* and *NIPAL4*, are indicated. The red line corresponds to a significance level of 2.5e-06 (5% adjusted with a Bonferroni correction for 20,000 genes).

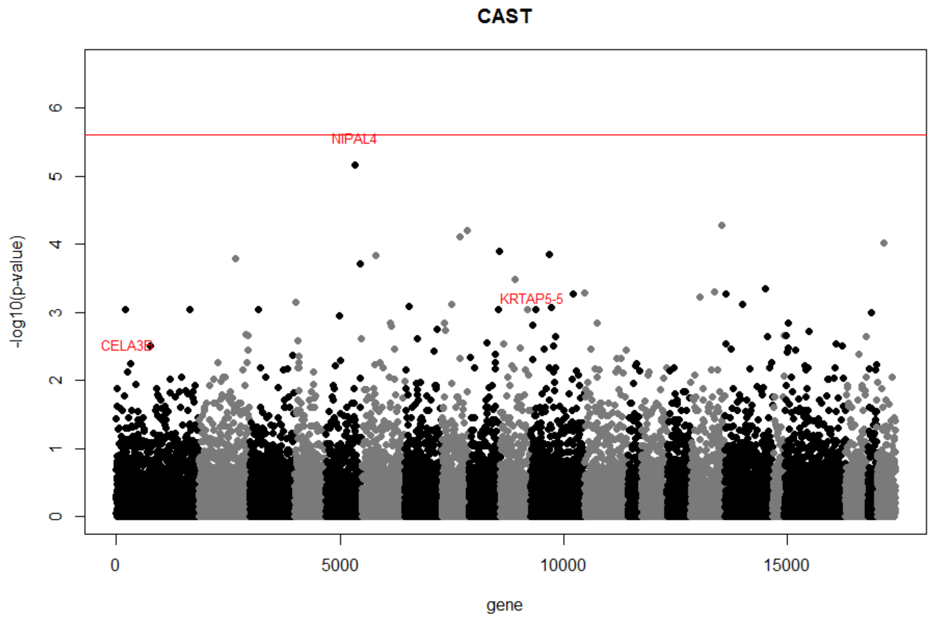

Fig A. Manhattan plot for EOAD results with CAST.

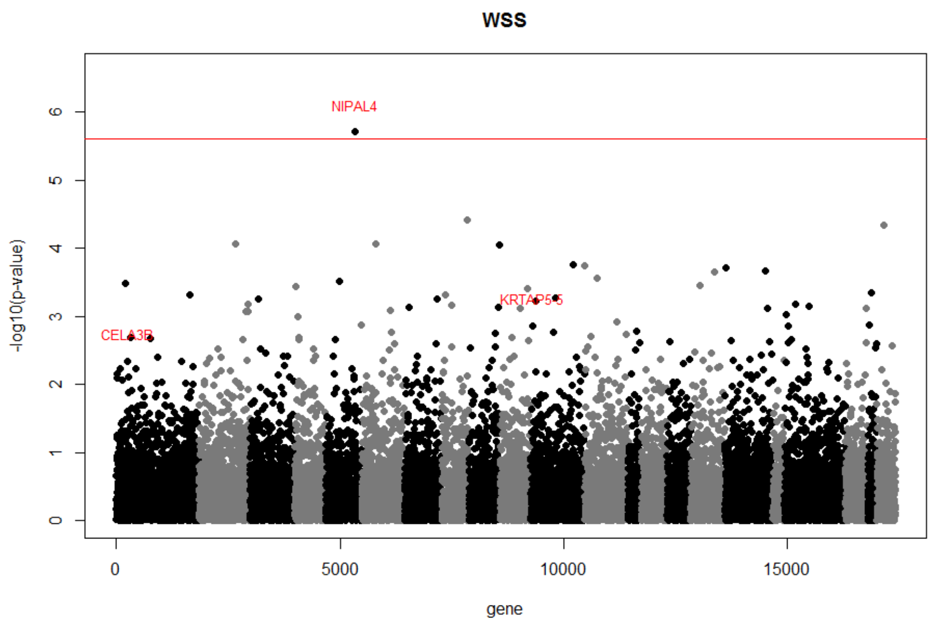

Fig B. Manhattan plot for EOAD results with WSS.

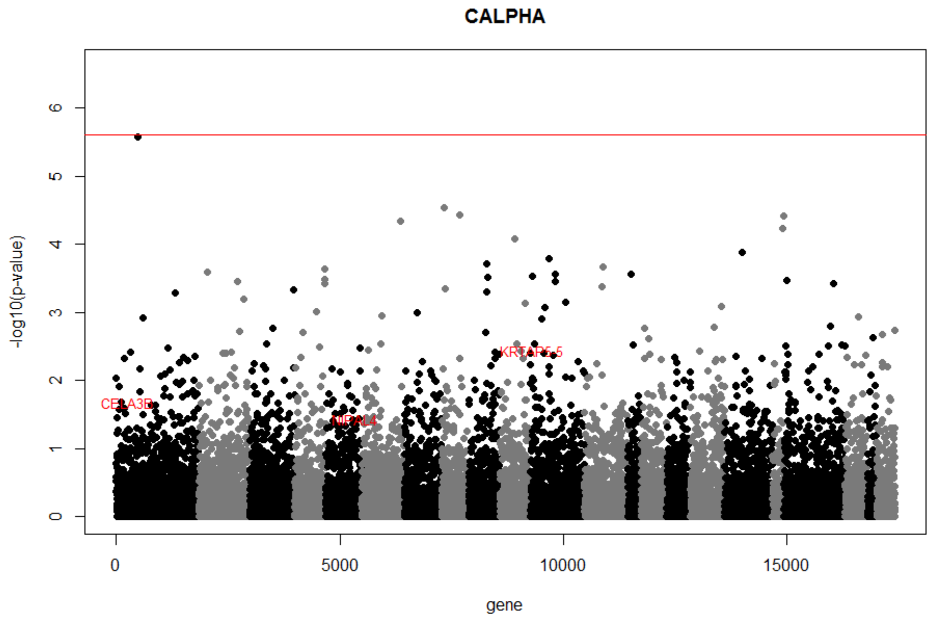

Fig C. Manhattan plot for EOAD results with C-alpha.

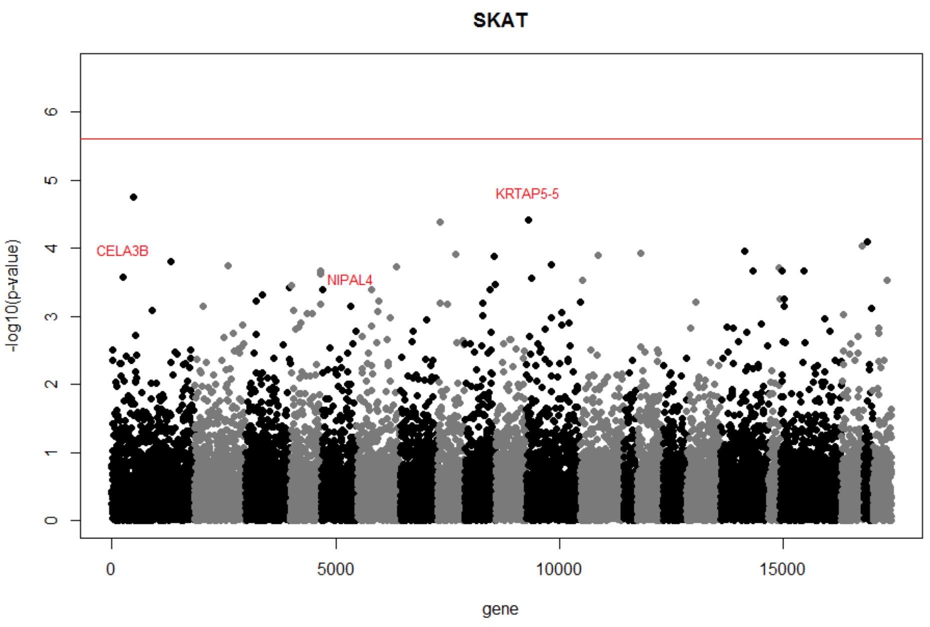

Fig D. Manhattan plot for EOAD results with SKAT.

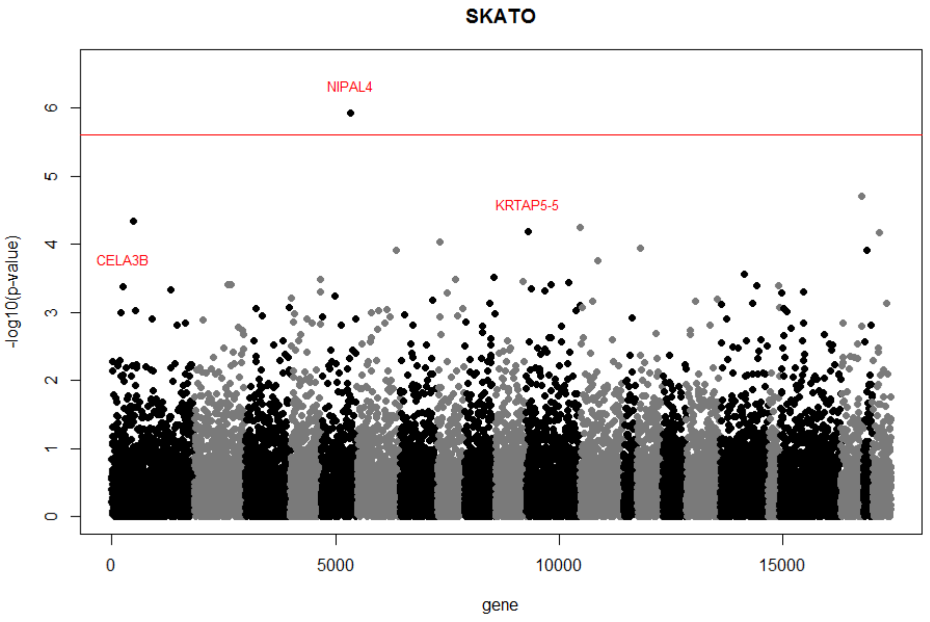

Fig E. Manhattan plot for SKAT-O results with WSS.

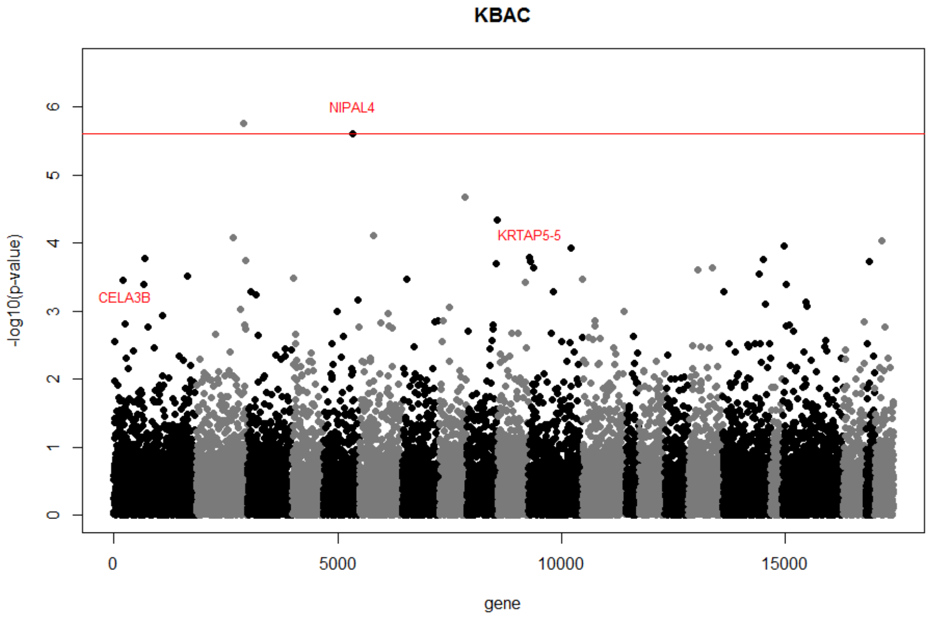

Fig F. Manhattan plot for EOAD results with KBAC.

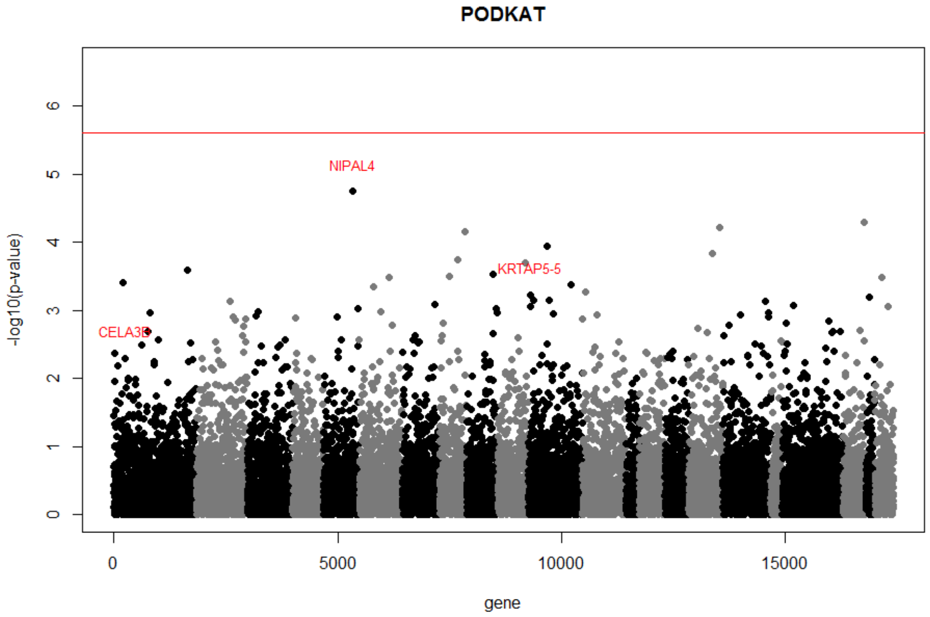

Fig G. Manhattan plot for EOAD results with PODKAT.

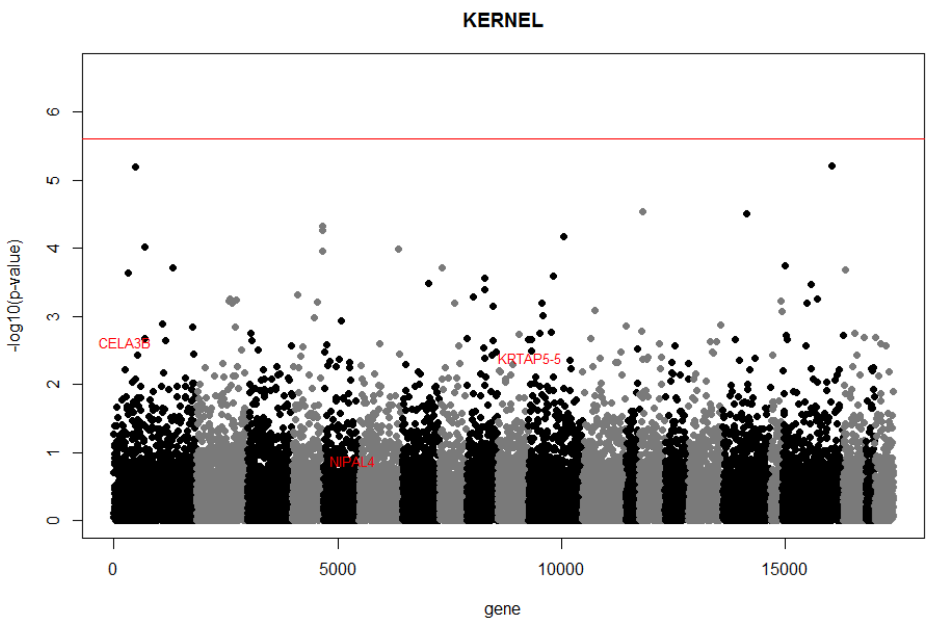

Fig H. Manhattan plot for EOAD results with KERNEL.

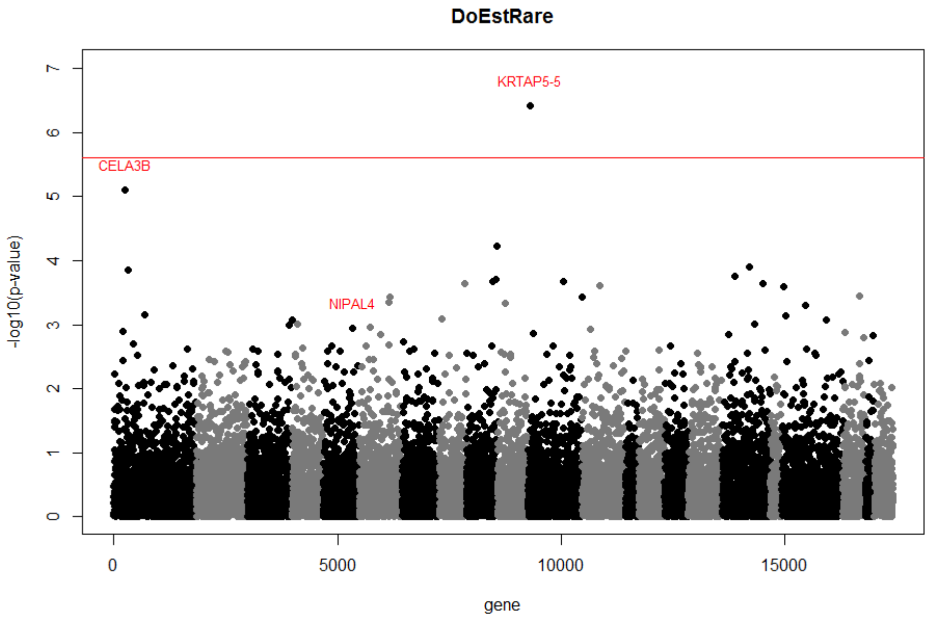

Fig I. Manhattan plot for EOAD results with DoEstRare.
