# Supplementary material for: DoEstRare: A statistical test to identify local enrichments in rare genomic variants associated with disease
Source: PLoS One. 2017 Jul 24;12(7):e0179364. doi: 10.1371/journal.pone.0179364 (PMC5524342; doi:10.1371/journal.pone.0179364)
Supplement: S3 Fig — From Fig A to Fig C, are represented allele counts and mutation position densities for the three genes, KRTAP5-5, CELA3B and NIPAL4, in cases and controls. Density functions for mutation positions were estimated with a Gaussian kernel. (PDF) [file pone.0179364.s004.pdf]

S3 Fig: Mutation position density plots for EOAD results

From Fig A to Fig C, are represented allele counts and mutation position densities for the three genes, *KRTAP5-5*, *CELA3B* and *NIPAL4*, in cases and controls. Density functions for mutation positions were estimated with a Gaussian kernel.

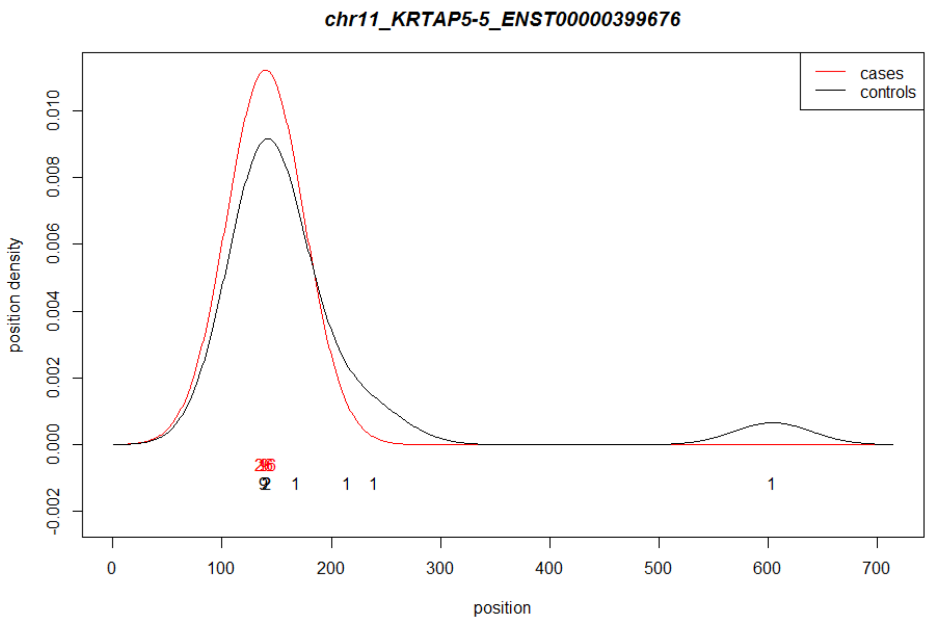

Fig A. Mutation position density for the *KRTAP5-5* gene (8 rare variants).

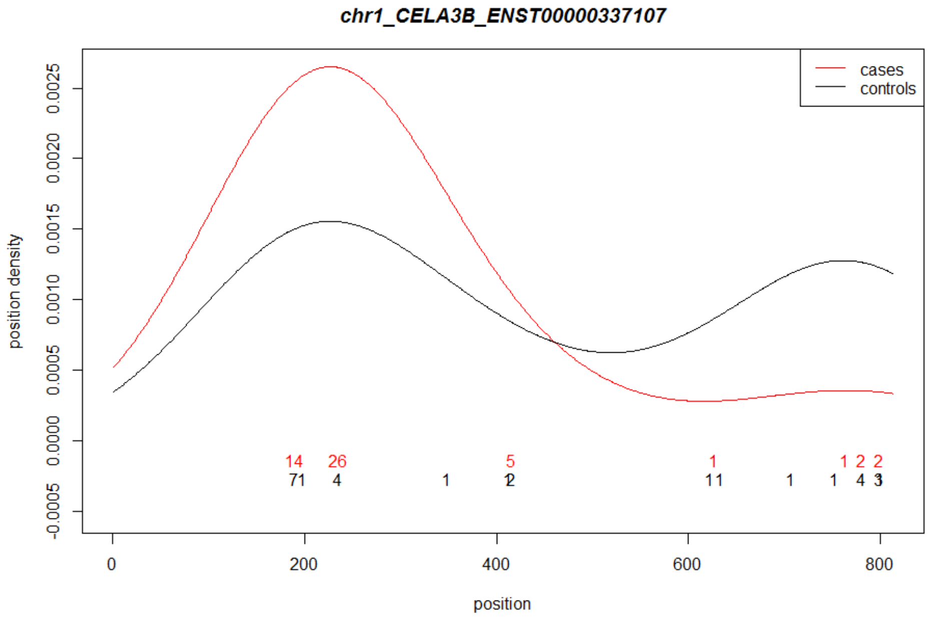

Fig B. Mutation position density for the *CELA3B* gene (15 rare variants).

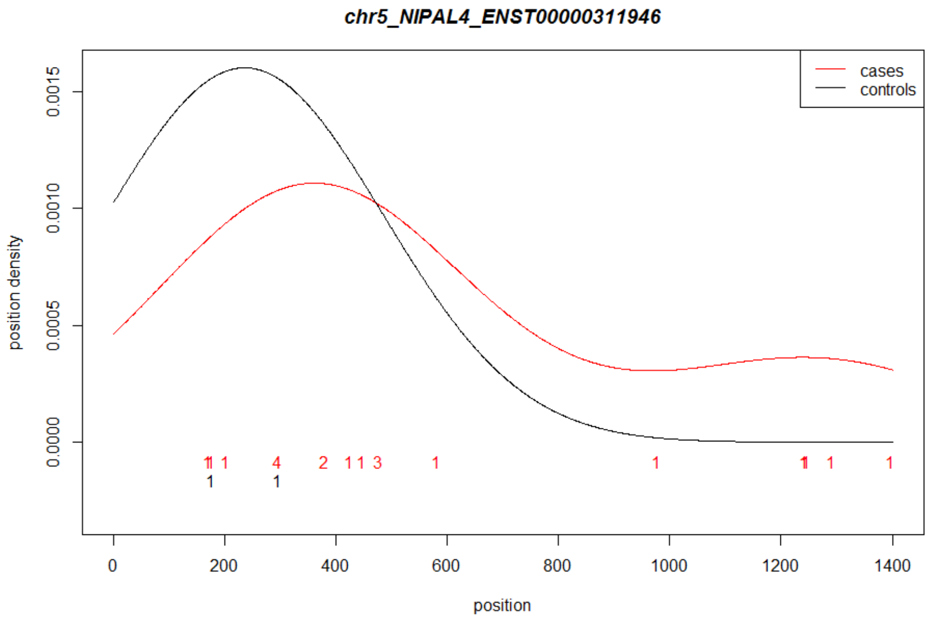

Fig C. Mutation position density for the *NIPAL4* gene (15 rare variants).
