# Supplementary material for: DoEstRare: A statistical test to identify local enrichments in rare genomic variants associated with disease
Source: PLoS One. 2017 Jul 24;12(7):e0179364. doi: 10.1371/journal.pone.0179364 (PMC5524342; doi:10.1371/journal.pone.0179364)
Supplement: S1 Text — Further details about the rare variant association tests we compared. (PDF) [file pone.0179364.s005.pdf]

S1 Text: Supplementary methods

Rare variant association tests

Notations

Let **X** be the matrix of genotypes with X_{ij} the count of rare alleles for the i-th individual and j-th rare variant, varying between 0, 1 or 2 rare alleles. Let $\boldsymbol{Y}$ be the vector of phenotypes with $Y_i = 1$ if the i-th individual is a case, $Y_i = 0$ if else. Let l_j be the position of the j-th rare variant. The number of affected (A) and unaffected (U) individuals are respectively N^A and N^U, with N the total number of individuals. The number of rare variants in the gene is P.

Methods not incorporating positional information

Our classification of association tests is partly based on the review from Lee et al. (2014) [1].

Burden tests

CAST

The "cohort allelic sum test" CAST described by Morgenthaler and Thilly in 2007 [2], uses a collapsing strategy, reducing the genetic information of a region to an indicator about the presence of rare mutations. We used a version comparing the proportions of individuals that present at least one mutation on a gene between cases and controls. The genetic score for the i-th individual is

$$C_{CAST_i} = I(S_i \geq 1)$$

with $S_i = \sum_{j=1}^{P} X_{ij}$, i.e. the sum of mutation counts on the gene for the i-th individual. A bilateral Fisher exact test is then applied between the binary genetic score $\boldsymbol{C_{CAST}}$ and the binary phenotype $\boldsymbol{Y}$.

WSS

The "weighted sum statistic" (WSS) developed by Madsen and Browning in 2009 [3] computes a genetic score per individual which is a weighted sum of mutations counts. Let the genetic score for the i-th individual be:

$$S_{WSS_i} = \sum_{j=1}^{P} w_{WSS_j} X_{ij}$$

The weight w_{WSS_j} is a continuous decreasing function of the control minor allele frequency:

$$w_{WSS_j} = \frac{1}{\sqrt{N.\widehat{MAF_j}^{U*}\left(1 - \widehat{MAF_j}^{U*}\right)}}$$

with $\widehat{MAF_j}^{U*} = \frac{\sum_{i=1}^{N^U} X_{ij}+1}{2(N^U+1)}$ the non-null estimator of the minor allele frequency in controls. Variants with a lower frequency present a more important weight so that they contribute equally to the genetic score under the null hypothesis. This also assumes that very rare variants are more likely to have an effect on disease susceptibility.

The association between the variables S_{WSS} and Y is tested with an unilateral Wilcoxon rank sum test, the null hypothesis being an excess of rare mutations in cases. The significance of the test can be evaluated by two different ways. The null hypothesis distribution of the test statistic can be approximated by a normal distribution, whose parameters are estimated by a phenotype permutation procedure. In this article we decided to evaluate the p-value with the empirical distribution obtained by permutations.

VT

The “variable threshold” (VT) approach developed by Price et al. in 2010 [4] supposes that rare variants with a frequency below a threshold t, which is determined adaptively, are more likely to be causal. A z-score noted $Z(t)$ is computed for each possible minor allele frequency threshold t:

$$Z(t) = \frac{\sum_{i=1}^{N}\sum_{j=1}^{P} I\left(\widehat{MAF_j} \le t\right) X_{ij}\,(Y_i - \bar{Y})}{\left[\sum_{i=1}^{N}\sum_{j=1}^{P}\left[I\left(\widehat{MAF_j} \le t\right) X_{ij}\right]^2\right]^{\frac{1}{2}}}$$

with $\widehat{MAF_j}$ the estimator of the minor allele frequency in the total population. The final test statistic is the maximum z-score $Z(t)$ across all possible t thresholds. In this article, the significance of the unilateral test is evaluated by a standard phenotype permutation procedure.

aSum

The “adaptive sum test” (aSum) developed by Han and Pan (2010) [5], computes a genetic score per individual in which weights take into account the direction of the genetic effect (protective or deleterious). The weight is equal to 1 ($w_{aSum_j} = 1$) for "deleterious-inclined" variants and is equal to -1 ($w_{aSum_j} = -1$) for "protective-inclined" variants. To decide whether the j-th variant is deleterious or protective inclined, the marginal logistic regression model is considered:

$$logit\left(P(Y_i = 1)\right) = \beta_0^{\mathrm{j}} + \beta_1^{j}\mathrm{X_{ij}}$$

If the estimation of the regression coefficient β_1^j is $\widehat{\beta_1^j} < 0$, and the p-value for the score test [6] is less than α_0, then the j-th variant is considered protective-inclined. We chose $\alpha_0 = 0.1$ as it is set in the original publication.

The aSum test is based on the following logistic regression model:

$$logit(P(Y_i = 1)) = \beta_0 + \beta_1 \mathrm{S}_{\mathrm{aSum}_i}$$

with $S_{aSum_i} = \sum_{j=1}^{P} w_{aSum_j} X_{ij}$ the genetic score for the i-th individual. The null hypothesis is $\beta_1 = 0$, which is tested with a score test [6]. We assessed the significance using the empirical distribution obtained by permutations.

P-value combination tests

ADA

The "adaptive combination of p-values for rare variant association testing" (ADA) developed by Lin et al. (2014) [7], is an extension of the sigma-P method [8] which is inspired from the Fisher method combining individual association signals. Rare variants are tested individually and the statistic is a weighted linear combination of p-value logarithms. Let $\{p_j, j \in \{1, \dots, P\}\}$ be the p-values obtained by a classical single-marker test. The test statistic Q_{ADA} is:

$$Q_{ADA} = -\sum_{j=1}^{P} w_{ADA_j} \log(p_j)$$

with w_{ADA_j} the weight and p_j the p-value for the j-th variant. This test uses the weight system proposed by Madsen and Browning (2009) [3] making the assumption that very rare variants play an important role. To deal with variants with opposite effects, it computes a statistic for "deleterious-inclined" variants and a statistic for "protective-inclined" variants. The weight for the ADA test is then:

$w_{ADA_j} = I(\widehat{MAF_j^A} \geq \widehat{MAF_j^U}) \times I(p_j \leq \theta) \times w_{WSS_j}$ for the "deleterious" statistic

$w_{ADA_j} = I(\widehat{MAF_j^A} < \widehat{MAF_j^U}) \times I(p_j \leq \theta) \times w_{WSS_j}$ for the "protective" statistic

with θ the p-value threshold which is determined to minimize the p-value of the ADA test. The final test statistic is the maximum of the two statistics. Contrary to the sigma-P method, the ADA test aims to effectively remove neutral variants from the analysis. Variants with a p-

value larger than a threshold θ are not taken into account in the analysis, the threshold being chosen adaptively. The significance is evaluated by a phenotype permutation procedure.

Remark: In the implementation of the ADA test, variants are tested individually with a Fisher exact test. Because of the discreteness of the hypergeometric distribution, p-values are particularly conservative in a context of rare variants, with an average greater than 0.5. This is why mid-p-values are computed [9].

Variance-component tests

The common assumption in burden tests is that causal variants have the same direction of effects; it means either protective or deleterious effect. A category of rare variant association tests, called **variance-component tests,** test unusual variance of minor allele frequencies in a group of variants.

C-alpha

The C-alpha test [10] has been developed to detect a mixture of genetic effects (neutral, protective or deleterious) in groups of variants. For a variant j-th, observed m_j times, we suppose that the number of mutations in cases m_j^A follow a binomial distribution $\mathcal{B}(m_j, p_j)$. Let p_0 be the proportion of cases in the dataset, under the null hypothesis, $\forall j \in \{1, \ldots, P\}$, $p_j = p_0$, i.e. a rare mutation occurs randomly in cases and controls. Under the alternative hypothesis the group of rare variants presents a mixture of binomial distributions with protective variants ($p_j < p_0$) or/and deleterious variants ($p_j > p_0$).

The C-alpha test is based on the principle that a mixture of binomial distributions leads to overdispersion. The statistic is then a sum of differences between observed variance and expected variance under the null hypothesis. Its expression is

$$Q_{C-alpha} = \sum_{j=1}^{P} \left[\left(m_j^A - m_j p_0 \right)^2 - m_j p_0 (1 - p_0) \right]$$

The significance is evaluated by a phenotype permutation procedure.

SKAT

The sequence kernel association test (SKAT) [11] is a generalization of the C-alpha test and uses a kernel matrix representing genetic similarities between individuals enabling more complex models. The logistic linear model is

$$logit\left(P(Y_i = 1|X_i)\right) = \beta_0 + \sum_{j=1}^{P} \beta_j X_{ij}$$

with β_0 the intercept and β_j, $j \in \{1, \ldots, P\}$ the regression coefficients for genetic effects. We consider in this model genetic factors as random effects. Each β_j follows an arbitrary distribution with a mean of zero and a variance of $w_j \tau$ with τ the variance-component and w_j the weight for the j-th variant. The null hypothesis in this test is H_0: $\tau = 0$. The statistic is

$$Q_{SKAT} = (\boldsymbol{Y} - \bar{Y})' \mathbf{K} (\boldsymbol{Y} - \bar{Y})$$

with **K** the kernel matrix corresponding to a genetic similarity matrix between individuals. In the context of the weighted linear model the kernel matrix is

$$\mathbf{K} = \mathbf{XWW'X'}$$

with $\mathbf{W} = diag\left(w_{SKAT_1}, \ldots, w_{SKAT_P}\right)$ where the weights are $w_{SKAT_j} = beta(MAF_j, a_1, a_2)$. Weights are function of the MAF, computed through the entire case-control sample. We chose to set the default values $a_1 = 1$, and $a_2 = 25$, as it increases the weight of very rare variants while still putting decent weights for less rare variants. The test statistic follows under the null distribution a mixture of chi-square distributions, which is approximated with the Davies

method [12], to assess the significance. For sample sizes strictly inferior to 2000, an adjustment is performed in the assessment of the p-value by approximated distribution [13].

SKAT-O

It has been noticed that the variance-component test SKAT is not powerful in a context of a high proportion of causal variants in a gene with the same effect [14]. That is why another version of SKAT called SKAT-O[13] has been developed with the strategy to optimally combine the variance-component test SKAT with a burden test. In this test, an additional parameter ρ, is adaptively determined to maximize the power. ρ is a coefficient between 0 and 1 that determines the correlation structure between genetic effects : $\forall j \; corr\left(\beta_j, \beta_{j'}\right) = \rho$. The statistic is

$$Q_{SKAT-O_\rho} = (\boldsymbol{Y} - \bar{Y})'\mathbf{K}_{\boldsymbol{\rho}}(\boldsymbol{Y} - \bar{Y})$$

with

$$\mathbf{K}_{\boldsymbol{\rho}} = \mathbf{XWR}_{\boldsymbol{\rho}}\mathbf{W}'\mathbf{X}'$$

where $\mathbf{R}_{\boldsymbol{\rho}} = (1-\rho)\boldsymbol{I_P} + \rho \boldsymbol{1_P}\boldsymbol{1_P}'$ and $\mathbf{W} = diag\left(w_{SKAT_1}, \dots, w_{SKAT_P}\right)$.

There are two particular cases:

- $\rho = 0$: $Q_\rho = \sum_{j=1}^{P} \mathrm{w}_j^2 \left[\sum_{i=1}^{N} (Y_i - \bar{Y}) X_{ij}\right]^2$ SKAT
- $\rho = 1$: $Q_\rho = \left[\sum_{j=1}^{P} \mathrm{w}_j^2 \sum_{i=1}^{N} (Y_i - \bar{Y}) X_{ij}\right]^2$ burden test

The coefficient ρ is determined among a series of values from 0 to 1 with an increment of 0.1 so that the p-value is minimal. The null distribution is approximated with the method described by Lee et al. (2012). For sample sizes strictly inferior to 2000, an adjustment is performed in the assessment of the p-value by approximated distribution [13].

The kernel-based adaptive cluster (KBAC) test

The kernel-based adaptive cluster (KBAC) test proposed by Liu and Leal (2010) [15] aims to overcome noise caused by neutral variants and gene interaction. It uses adaptive weights to better discriminate multi-site genotypes. A more important weight is accorded to the multi-site genotypes (or mutations patterns) that are enriched in cases.

Suppose that X_{KBAC_l}, $l \epsilon \{0, \dots, L\}$, are the different multi-site genotypes we observe in our dataset **X**, and that n_l, $l\{0, \dots, L\}$ are the observed counts for each different mutation pattern. The KBAC statistic is given by

$$KBAC = \left(\sum_{l=1}^{L} w_l \left(\frac{n_l^A}{N^A} - \frac{n_l^U}{N^U} \right) \right)^2$$

with w_l the weight for l-th multi-site genotype which is computed adaptively with the choice of a kernel function. The hypergeometric kernel is the most often used in the literature and is suitable for small to moderate sample sizes. It is assumed that under the null hypothesis the count N_l^A of the l-th pattern in cases follows an hypergeometric distribution $\mathcal{H}\left(N^A, \frac{n_l}{N}, N\right)$. The weight w_l for the l-th multi-site genotype is defined as

$$w_l = P(N_l^A \geq n_l^A) = \sum_{k=0}^{n_l^A} \frac{\binom{N_l}{k}\binom{N - N_l}{N^A - k}}{\binom{N}{N^A}}$$

The significance of the KBAC test is evaluated by a phenotype permutation procedure.

Methods that incorporate positional information

Few tests take into account physical positions of rare variants. There are two main strategies to detect associated clustered rare variants: sliding windows and distance matrix.

Tests using sliding windows

Burden or Mutation Position test (BOMP)

A simple approach to detect a cluster of DRVs in a gene is to compute a statistic per window of the gene. Because the size and the location of the cluster are usually unknown, sliding windows of different sizes are commonly considered. This strategy is used in the tests Burden or Mutation Position (BOMP) test [16], proposed by Chen et al. in 2013, and the test developed by Ionita-Laza et al. in 2012 [17].

The BOMP test [16] consists in a combination of two likelihood ratio tests: a burden test and a mutation position distribution test.

The burden test uses an approach similar to CAST, and the collapsing variable is

$$C_i = I(S_i \geq t)$$

with t a threshold that is determined to maximize the test statistic. For the computation of this log-likelihood ratio statistic $\Lambda_{burden}(t)$, it is assumed that presenting at least t rare mutations follows a Bernoulli distribution of success probability p^A, p^U or p^{A+U} for case, control or total population. Under the null hypothesis, these probabilities are equal. The final statistic Λ_{burden} is given for the threshold t that maximizes the function $\Lambda_{burden}(t)$, i.e. $\Lambda_{burden} = \max_t\big(\Lambda_{burden}(t)\big)$.

For the mutation position test, the gene is divided into M windows. The window mutations counts follow a multinomial distribution of parameters $p_m^A, m \in \{1, \dots, M\}$ for cases, $p_m^U, m \in \{1, \dots, M\}$ for controls and $p_m^{A+U}, m \in \{1, \dots, M\}$ for the total population. Under the null hypothesis the parameters are equal for each multinomial distribution. The log-likelihood ratio statistic $\Lambda_{position}$ is computed for several partitions of the gene by a sliding window procedure with different window sizes. The statistic is the maximum statistic over the different partitions.

The final BOMP statistic is the sum of the log-likelihood ratio statistics obtained for the two tests

$$\Lambda_{BOMP} = \Lambda_{burden} + \Lambda_{position}$$

The significance of the total BOMP statistic is evaluated by a phenotype permutation procedure.

Tests using kernel matrix

KERNEL

The test proposed by Schaid et al. in 2013 [18], is inspired from the Tango spatial method[19] which aims to detect spatial disease clusters. Let $\boldsymbol{\delta_{Kernel}}$ be the vector of difference in minor allele frequencies between cases and controls. The value of one element of this vector is

$$\delta_{Kernel_j} = \frac{m_j^A}{\sum_{j=1}^{P} m_j^A} - \frac{m_j^U}{\sum_{j=1}^{P} m_j^U}$$

with m_j^A and m_j^U the counts of rare mutations for the j-th variant in cases and controls respectively. Position information is contained in a kernel matrix $\mathbf{A}$ that measures distances between pairs of rare variants. The quadratic test statistic is then

$$Q_{Kernel} = \boldsymbol{\delta}'_{\boldsymbol{Kernel}} \mathbf{A} \boldsymbol{\delta}_{\boldsymbol{Kernel}}$$

The kernel function is the tri-weight

$$A_{jj'}(c) = K\left(d'_{jj'}(c)\right) = \left(1 - d'_{jj'}(c)^2\right)^3$$

with $d'_{jj'}(c) = \frac{d_{jj'}}{c \times maxd}$ where $d_{jj'} = \left|l_{j'} - l_j\right|$ is the physical distance between variants j and j' ; $maxd$ is a user-specified maximum distance (gene length or coding length); c is a coefficient varying from 0.1 to 1 with an increment of 0.1. The final test statistic is the

maximum over the computed statistics with the ten different values of c. The significance is evaluated by a phenotype permutation procedure.

CLUSTER

The test CLUSTER [20], proposed by Lin in 2014, is an extension of the test ADA[7] which has been described previously and is inspired from the test developed by Schaid et al. [18]. In a vector $\boldsymbol{\delta}_{\boldsymbol{CLUSTER}}$, are indicated the weighted logarithms of single-marker p-values. The value for one element of this vector is

$$\delta_{CLUSTER_j} = \sqrt{-w_{ADA_j} log(p_j)}$$

The quadratic test statistic is then

$$Q_{CLUSTER} = \boldsymbol{\delta}'_{\boldsymbol{CLUSTER}} \mathbf{A} \boldsymbol{\delta}_{\boldsymbol{CLUSTER}}$$

with **A** the same kernel matrix as for the KERNEL test. Like the test ADA, two statistics are computed for “deleterious-inclined” variants and for “protective-inclined” variants; and the final statistic is the maximum of these two statistics. The significance is evaluated by a phenotype permutation procedure.

Position-Dependent Kernel Association Test (PODKAT)

The test PODKAT, has been proposed by Bodenhofer [21], is an extension of the test SKAT. The test statistic is similar to the SKAT test statistic:

$$Q_{PODKAT} = (\boldsymbol{Y} - \bar{Y})'\mathbf{K}(\boldsymbol{Y} - \bar{Y})$$

where the position-dependent linear kernel is :

$$\mathbf{K} = \mathbf{XWAA'W'X'}$$

The kernel matrix **K** incorporates the SKAT weight matrix $\mathbf{W} = diag(w_{SKAT_1}, \dots, w_{SKAT_P})$ and a position-dependent matrix **A** measuring similarities/closeness of positions of variants:

$$A_{j,j'} = \max\left(1 - \frac{1}{w} d_{j,j'}, 0\right)$$

The parameter w is called "maximal radius of tolerance", by default its value is 1000 bp. By analogy with KERNEL distance measure $d'_{jj'}(c)$, the parameter w is then equivalent to $c \times maxd$, with $c = 0.1$ in our simulation context as $maxd = 10$ kb.

The distance-based measure (DBM) test

The test developed by Fier et al. [22] compares two weighted distance distribution functions in cases and controls with the Ansari-Bradley test [23].

Let $\boldsymbol{S^A}$ and $\boldsymbol{S^U}$ be the variant position sequences for cases and controls. In these vectors are incorporated variant positions, which are repeated $[m_j^A w_j]$ in cases or $[m_j^U w_j]$ in controls (round numbers) to account spatial distribution of allele frequencies. Then allele distances are derived in $\boldsymbol{D^A}$ and $\boldsymbol{D^U}$ vectors, by subtracting two consecutive elements of vectors $\boldsymbol{S^A}$ and $\boldsymbol{S^U}$. An Ansari-Bradley test is applied to $\boldsymbol{D^A}$ and $\boldsymbol{D^U}$ vectors to compare the weighted distance distributions in cases and controls.

The weighted scheme adopted by Fier et al. [22] depends of minor allele frequencies and distance to the closest neighbour. Two kinds of weight are computed according to the distribution of variants in cases or controls. Weights, based on the distribution of variants in cases are:

$$w_j^A = 1 + \frac{\frac{m^A + 1}{m_j^A + 1}}{\log\left(d_{min_j} + 1\right)}$$

with d_{min_j} the distance between the j-th variant and its closest neighbour. In the same way, weights based on the distribution of variants in cases are:

$$w_j^U = 1 + \frac{\frac{m^U + 1}{m_j^U + 1}}{\log\left(d_{min_j} + 1\right)}$$

A test statistic is computed for each weighted scheme and the final test statistic corresponds to the maximum. The significance is finally evaluated by a phenotype permutation procedure.

Implementation of the different statistical tests

Test	Source type	Source link	Arguments other than default
CAST	R package AssotesteR (CRAN)	https://cran.r-project.org/src/contrib/AssotesteR_0.1-10.tar.gz	maf=0.5<br>(variants already filtered on the MAF)
WSS	R code		
VT	R code		
aSum	R code from Wei Pan's website	http://www.biostat.umn.edu/~weip/prog/BasuPanGE11/aSumTest.r	alpha0=0.1
C-ALPHA	R code based on Wei Pan's code	http://www.biostat.umn.edu/~weip/prog/BasuPanGE11/CalphaP.R	
SKAT	R package SKAT (CRAN)	https://cran.r-project.org/src/contrib/SKAT_1.1.2.tar.gz	
SKAT-O	R package SKAT (CRAN)	https://cran.r-project.org/src/contrib/SKAT_1.1.2.tar.gz	method="optimal"
KBAC	R package KBAC	http://tigerwang.org/software/kbac	alpha = 999<br>(to not use the KBAC adaptive p-value calculation)
ADA	R code from Wan-Yu Lin's website	http://homepage.ntu.edu.tw/~linwy/ADA.html	mafThr = 0.5
DBM	R code sent by authors		
CLUSTER	R code from Wan-Yu Lin's website	http://homepage.ntu.edu.tw/~linwy/CLUSTER.html	mafThr = 0.5<br>max_d=$maxd$
KERNEL	R code		
PODKAT	R code on Bioconductor	https://www.bioconductor.org/packages/release/bioc/src/contrib/podkat_1.2.0.tar.gz	kernel="linear.podkat"
BOMP	Software java	http://karchinlab.org/apps/appBomp.html	

POWER COMPARISON BETWEEN SIMULATED SCENARIOS

For each statistical test, we wanted to compare the differences in power at α=5% between the simulated scenarios 1 and 2, which correspond respectively to situations with no clustered DRVs and one cluster of DRVs. We performed a Fisher exact test on the following 2x2 contingency table, to test the independency between the significant/non-significant status of the gene and the simulated scenario.

	Scenario 1	Scenario 2
Significant gene at α=5%	a	b
Non-significant gene at α=5%	c	d
Total	1000 replicates	1000 replicates

We also compared scenarios 2 and 3, to see if there was a power difference between one and two clusters of DRVs.

REFERENCES

1. Lee S, Abecasis GR, Boehnke M, Lin X. Rare-Variant Association Analysis: Study Designs and Statistical Tests. Am. J. Hum. Genet. 2014;95:5–23.

2. Morgenthaler S, Thilly WG. A strategy to discover genes that carry multi-allelic or mono-allelic risk for common diseases: a cohort allelic sums test (CAST). Mutat. Res. 2007;615:28–56.

3. Madsen BE, Browning SR. A groupwise association test for rare mutations using a weighted sum statistic. PLoS Genet. 2009;5:e1000384.

4. Price AL, Kryukov GV, de Bakker PIW, Purcell SM, Staples J, Wei L-J, et al. Pooled association tests for rare variants in exon-resequencing studies. Am. J. Hum. Genet. 2010;86:832–8.

5. Han F, Pan W. A data-adaptive sum test for disease association with multiple common or rare variants. Hum. Hered. 2010;70:42–54.

6. Clayton D, Chapman J, Cooper J. Use of unphased multilocus genotype data in indirect association studies. Genet. Epidemiol. 2004;27:415–28.

7. Lin W-Y, Lou X-Y, Gao G, Liu N. Rare variant association testing by adaptive combination of P-values. PloS One. 2014;9:e85728.

8. Cheung YH, Wang G, Leal SM, Wang S. A fast and noise-resilient approach to detect rare-variant associations with deep sequencing data for complex disorders. Genet. Epidemiol. 2012;36:675–85.

9. Armitage P, Berry G, Matthews JNS. Statistical Methods in Medical Research. 4th Edition. Blackwell Publishing; 2002.

10. Neale BM, Rivas MA, Voight BF, Altshuler D, Devlin B, Orho-Melander M, et al. Testing for an Unusual Distribution of Rare Variants. PLoS Genet. 2011;7:e1001322.

11. Wu MC, Lee S, Cai T, Li Y, Boehnke M, Lin X. Rare-Variant Association Testing for Sequencing Data with the Sequence Kernel Association Test. Am. J. Hum. Genet. 2011;89:82–93.

12. Davies RB. Algorithm AS 155: The Distribution of a Linear Combination of $\chi 2$ Random Variables. J. R. Stat. Soc. Ser. C Appl. Stat. 1980;29:323–33.

13. Lee S, Emond MJ, Bamshad MJ, Barnes KC, Rieder MJ, Nickerson DA, et al. Optimal Unified Approach for Rare-Variant Association Testing with Application to Small-Sample Case-Control Whole-Exome Sequencing Studies. Am. J. Hum. Genet. 2012;91:224–37.

14. Basu S, Pan W. Comparison of statistical tests for disease association with rare variants. Genet. Epidemiol. 2011;35:606–19.

15. Liu DJ, Leal SM. A Novel Adaptive Method for the Analysis of Next-Generation Sequencing Data to Detect Complex Trait Associations with Rare Variants Due to Gene Main Effects and Interactions. PLoS Genet. 2010;6:e1001156.

16. Chen Y-C, Carter H, Parla J, Kramer M, Goes FS, Pirooznia M, et al. A hybrid likelihood model for sequence-based disease association studies. PLoS Genet. 2013;9:e1003224.

17. Ionita-Laza I, Makarov V, ARRA Autism Sequencing Consortium, Buxbaum JD. Scan-statistic approach identifies clusters of rare disease variants in LRP2, a gene linked and associated with autism spectrum disorders, in three datasets. Am. J. Hum. Genet. 2012;90:1002–13.

18. Schaid DJ, Sinnwell JP, McDonnell SK, Thibodeau SN. Detecting genomic clustering of risk variants from sequence data: cases versus controls. Hum. Genet. 2013;132:1301–9.

19. Tango T. Statistical Methods for Disease Clustering. Springer, New York; 2010.

20. Lin W-Y. Association testing of clustered rare causal variants in case-control studies. PloS One. 2014;9:e94337.

21. Bodenhofer U. PODKAT: An R Package for Association Testing Involving Rare and Private Variants. R package version 1.0.3; 2015.

22. Fier H, Won S, Prokopenko D, AlChawa T, Ludwig KU, Fimmers R, et al. “Location, Location, Location”: a spatial approach for rare variant analysis and an application to a study on non-syndromic cleft lip with or without cleft palate. Bioinforma. Oxf. Engl. 2012;28:3027–33.

23. Ansari AR, Bradley RA. Rank-Sum Tests for Dispersions. Ann. Math. Stat. 1960;31:1174–89.
